# Oral inulin prebiotic encapsulating *Cortex Fraxini* extracellular vesicles alleviates colitis and associated renal stress via reinforcing intestinal barriers and remodeling microbiota

**DOI:** 10.7150/thno.139484

**Published:** 2026-09-02

**Authors:** Meihong Chai, Junlong Fu, Mengjie Zhang, Xing Pei, Xingzhou Liu, Bing Xiao, Bowen Gao, Jingya Wei, Baosen Yue, Mingxin Zhang, Mingzhen Zhang, Juan Jin

**Affiliations:** 1 Department of Pharmacy, Xi’an Hospital of Traditional Chinese Medicine, Xi’an, Shaanxi, 710021, China.; 2 Department of Pharmacology, School of Basic Medical Sciences, Xi’an Jiaotong University, Xi’an, Shaanxi, 710061, China.; 3 School of Life Science; School of Interdisciplinary Science; Aerospace Center Hospital; Key Laboratory of Molecular Medicine and Biotherapy; Key Laboratory of Medical Molecule Science and Pharmaceutics Engineering; Beijing Institute of Technology, Beijing, 100081, China.; 4 Tianjin Key Laboratory of Food and Biotechnology, School of Biotechnology and Food Science, Tianjin University of Commerce, Tianjin, 300134, China.; 5 Department of Gastroenterology, The First Affiliated Hospital of Xi’an Medical University, Xi’an, Shaanxi, 710077, China.; 6 Department of Nephrology, the First Affiliated Hospital of Zhejiang Chinese Medical University (Zhejiang Provincial Hospital of Chinese Medicine), Hangzhou, Zhejiang, 310003, China.

**Keywords:** *Cortex Fraxini*-derived extracellular vesicles, inulin hydrogel, ulcerative colitis, gut-kidney axis, intestinal barrier

## Abstract

**Background: Gut–kidney axis dysfunction contributes to extraintestinal complications in inflammatory bowel disease (IBD), yet effective therapeutic strategies targeting this axis remain limited. This study developed an oral prebiotic delivery system by encapsulating *Cortex Fraxini*-derived extracellular vesicles (CF-EVs) within an inulin-based hydrogel (CF-EVs@Inulin) and evaluated its therapeutic potential in ulcerative colitis and associated renal injur**y.

**Methods: CF-EVs were isolated and characterized for morphology and antioxidant activity. An inulin-based hydrogel was employed to encapsulate CF-EVs and improve gastrointestinal stability and colonic retention. Therapeutic efficacy was assessed in dextran sulfate sodium (DSS)-induced acute colitis mice, including antibiotic-pretreated groups. Gut microbiota composition, intestinal barrier integrity, intestinal permeability, lipopolysaccharide (LPS) translocation, renal TLR4/NF-κB signaling, and renal injury markers were** analyzed.

**Results: CF-EVs exhibited typical vesicular morphology and potent ROS-scavenging activity. CF-EVs@Inulin showed enhanced gastrointestinal stability and prolonged retention in the colon. Treatment with CF-EVs@Inulin significantly ameliorated colitis symptoms, restored mucus and tight junction barriers, and remodeled gut microbiota composition. These protective effects were largely attenuated following microbiota depletion. Mechanistically, CF-EVs@Inulin reduced intestinal permeability and circulating LPS levels, suppressed renal TLR4/NF-κB activation, improved renal function, and decreased the expression of kidney injury markers KIM-1 and** NGAL.

**Conclusion: CF-EVs@Inulin alleviates colitis-associated early renal stress by restoring intestinal barrier integrity and gut microbiota homeostasis, thereby interrupting the LPS–TLR4/NF-κB signaling pathway. These findings highlight the therapeutic potential of prebiotic-encapsulated CF-EVs as a promising strategy for ulcerative colitis and gut–kidney axis-related com**plications.

## Introduction

In recent years, the concept of the “organ axis” has reshaped our understanding of disease pathogenesis, highlighting the dynamic crosstalk among different organs through metabolic, immune, and neuroendocrine signaling networks 1, 2. Among these interconnected systems, the gut has emerged as a central hub due to its extensive microbiota and complex immune interface 3, 4. In 2011, Meijers et al. first proposed the concept of the gut-kidney axis 5. Accumulating evidence indicates that gut microbial dysbiosis contributes to the pathogenesis and progression of chronic kidney disease (CKD), primarily through the accumulation of uremic toxins and the promotion of chronic systemic inflammation. Subsequent studies further revealed that microbial imbalance not only reduces beneficial metabolites such as short-chain fatty acids (SCFAs) 6, 7, but also increases the production of harmful metabolites, including indoxyl sulfate (IS), p-cresyl sulfate (pCS) 8, 9, and trimethylamine N-oxide (TMAO) 10, 11. These gut-derived toxins can translocate into the systemic circulation through a disrupted intestinal barrier and accumulate in the kidney, where they activate the Toll-like receptor 4/nuclear factor-κB (TLR4/NF-κB) pathway and the renin-angiotensin system (RAS) 12, while simultaneously inducing oxidative stress 13, 14. Collectively, these events establish a vicious cycle of “microbiota dysbiosis-barrier disruption-toxin translocation-renal injury”. However, current studies have mainly focused on the deterioration of the gut-kidney axis in CKD, whereas the early renal stress induced by intestinal inflammation and its upstream intervention strategies remain insufficiently explored.

Inflammatory bowel disease (IBD), mainly comprising Crohn’s disease (CD) and ulcerative colitis (UC) 15, is characterized by chronic relapsing intestinal inflammation. UC is pathologically characterized by continuous mucosal inflammation in the colon and is associated with genetic susceptibility, immune dysregulation, environmental factors, and gut microbiota imbalance 16-18. Increasing evidence has identified intestinal barrier dysfunction as a critical pathological basis of IBD 19-21. Under inflammatory conditions, excessive ROS generated by oxidative stress directly damages intestinal epithelial cells and tight junction proteins 22. Meanwhile, gut dysbiosis is characterized by a reduction in beneficial commensal bacteria such as *Faecalibacterium prausnitzii* 23, 24, an increase in opportunistic pathogens, and decreased levels of protective metabolites such as butyrate 25. In addition, bacterial-derived LPS further amplifies inflammatory responses through activation of the TLR4/NF-κB signaling pathway 26. Barrier disruption consequently increases intestinal permeability 27, facilitating the translocation of bacterial toxins and antigens into the circulation, thereby aggravating local intestinal inflammation and contributing to extraintestinal organ injury 28, 29. Notably, approximately one-quarter of IBD patients develop renal complications, including CKD, glomerulonephritis, and tubulointerstitial diseases 30-33. Nevertheless, therapeutic strategies targeting intestinal barrier restoration to interrupt early renal stress signaling along the gut-kidney axis remain largely underexplored in UC research.

As a traditional Chinese herbal medicine, *Cortex Fraxini* has been used for the treatment of dysentery and intestinal inflammatory diseases for a long time. It contains rich biologically active ingredients, including coumarins, iridoids, and phenylethanoid glycosides 34. Among them, *fraxin* has been shown to reduce DSS-induced colitis by inhibiting the generation of reactive oxygen species (ROS), reducing the release of pro-inflammatory cytokines, and regulating the TLR4/NF-κB and MAPK signaling pathways 35. However, the low oral bioavailability of these bioactive ingredients limits their clinical transformation and application. In recent years, plant-derived extracellular vesicles (PDEVs) have received increasing attention as natural nanocarriers 36, 37. PDEVs have a lipid bimolecular structure, can carry proteins, RNA, and bioactive phytochemicals, and naturally have cross-species intestinal absorption and immunomodulation characteristics 38. Notably, PDEVs have demonstrated promising anti-inflammatory and microbiota-regulating activities in UC models 39. However, despite their promising therapeutic potential, orally administered CHM-EVs still face several limitations, including poor stability in the gastrointestinal tract, insufficient colonic retention, and limited interactions with the gut microbiota, which may compromise their therapeutic efficacy. Inulin, a well-recognized prebiotic, selectively promotes the proliferation of beneficial bacteria such as *Bifidobacterium* and *Lactobacillus* and enhances intestinal barrier integrity through microbial fermentation-derived SCFAs 40. Inulin-based hydrogels further integrate colon-targeted delivery with prebiotic functionality. They remain stable in the stomach and small intestine, but undergo specific degradation by microbial inulinase upon reaching the colon, enabling localized sustained drug release while synergistically promoting microbiota remodeling and barrier repair 41.

Based on these considerations, extracellular vesicles derived from *Cortex Fraxini* (CF-EVs) were isolated and encapsulated within an inulin hydrogel to construct an oral **prebiotic** delivery system (CF-EVs@Inulin) with integrated functions of colon-targeted sustained release, intestinal barrier repair, and gut microbiota modulation. In a DSS-induced acute UC model, the therapeutic effects and mechanisms of CF-EVs@Inulin in alleviating intestinal inflammation and early renal stress were systematically investigated **(Figure 1)**.

## Results

### Isolation and characterization of CF-EVs

CF-EVs were isolated and purified from dried *Cortex Fraxini* using differential ultracentrifugation combined with sucrose density gradient centrifugation **(Figure 2A)**. Following density gradient separation, fractions corresponding to different sucrose concentrations were collected separately. CF-EVs were found to be predominantly enriched in the 30 %-45 % sucrose density fractions** (Figure 2B)**. Transmission electron microscopy (TEM) revealed that the vesicles in these fractions exhibited a typical spherical morphology with well-defined boundaries and an intact lipid bilayer membrane structure **(Figure 2C)**. Dynamic light scattering (DLS) analysis shows that the average hydration particle size of CF-EVs is 157.4 ± 60.02 nm** (Figure 2D)**, and the Zeta potential is -34.7 ± 6.47 mV **(Figure 2E)**. Its negatively charged surface helps to maintain good colloidal stability in aqueous solution 42. Overall, the observed morphology, particle size distribution, and surface charge characteristics are consistent with the typical characteristics of extracellular vesicles, indicating that CF-EVs with relatively high purity have been successfully obtained, which can be used for subsequent encapsulation and functional research of inulin hydrogel. Given that oxidative stress plays a key role in the pathogenesis of ulcerative colitis and its related intestinal barrier dysfunction, the antioxidant potential of CF-EVs was studied.

To systematically evaluate the antioxidant activity of CF-EVs, the ABTS^+^ free radical clearance experiment, the TMB-based hydroxyl radical (•OH) removal experiment, the PTIO single-line oxygen clearance experiment, and the DPPH free radical removal experiment were first tested. As shown in** Figures 2F-I**, CF-EVs show concentration-dependent free radical clearance activity in the concentration range of 12.5-100 μg/mL. At a concentration of 100 μg/mL, the clearance rate of CF-EVs for ABTS^+^ free radicals are about 70 %** (Figure 2F)**, the clearance rate of •OH free radicals are about 80 % **(Figure 2G)**, the clearance rate of DPPH free radicals is about 55 % **(Figure 2H)**, and the clearance rate of PTIO single-line oxygen is about 50 % **(Figure 2I)**, showing a broad spectrum and significant antioxidant activity. To further verify its removal efficiency of specific reactive oxygen species, the removal effect of CF-EVs on ABTS cationic free radicals (ABTS•^+^), •OH, PTIO free radicals, and DPPH free radicals (DPPH•) was directly evaluated by the electron paramagnetic resonance (EPR) spectrum method **(Figures 2J-M)**. The EPR spectrum confirms that CF-EVs can effectively quench a variety of free radical species in a concentration-dependent manner, further proving their broad-spectrum antioxidant activity. The above results show that CF-EVs have been successfully isolated, with typical extracellular vesicle characteristics and inherent broad-spectrum antioxidant activity, suggesting that CF-EVs are expected to be used as a promising bioactive nanoplatform for the treatment of oxidative stress-driven intestinal diseases.

To further explore the molecular basis underlying the antioxidant properties of CF-EVs, we characterized the biomolecular cargos carried by CF-EVs. SDS-PAGE followed by Coomassie brilliant blue staining and agarose gel electrophoresis revealed that CF-EVs contain abundant protein and nucleic acid components **(Figures S1A-B)**. Furthermore, LC-MS/MS-based untargeted metabolomic analysis was performed to characterize the small-molecule cargos associated with the antioxidant activity of CF-EVs. Multiple plant-derived metabolites were identified, including representative coumarin derivatives (*esculetin, fraxetin, aesculin*, and *fraxin*) as well as phenolic compounds with reported antioxidant properties. These bioactive phytochemicals, together with the identified protein and nucleic acid cargos, may collectively contribute to the ROS-scavenging capacity of CF-EVs **(Figures S1C-D)**.

### Protective effects of CF-EVs against oxidative stress and inflammation in cells

The primary objective of this study was to determine whether inulin, as a prebiotic, could indirectly restore intestinal barrier integrity and mitigate early renal stress by modulating the gut microbiota. Because conventional *in vitro* culture systems lack microbial communities and therefore fail to recapitulate microbiota-dependent ecological interactions, the direct biological effects of inulin could not be adequately assessed under these conditions. Moreover, inulin has previously been reported to exhibit only limited antioxidant and anti-inflammatory activity in isolated cell models 43, 44. Its incorporation into hydrogel formulations also substantially alters viscosity and rheological behavior, while the sustained-release properties central to its function are difficult to model in static cell culture systems and may interfere with cellular assays. Accordingly, the *in vitro* experiments were designed to evaluate the intrinsic biological activities of CF-EVs specifically.

To investigate the cellular functions of CF-EVs, their anti-inflammatory activity was first examined in RAW 264.7 murine macrophages. Since efficient intracellular delivery is a key prerequisite for vesicle-mediated biological activity, the uptake behavior of CF-EVs was first characterized. Fluorescence microscopy and flow cytometry were used to monitor the time-dependent uptake of Dil-labeled CF-EVs in RAW 264.7 cells. As shown in **Figure 3A**, CF-EVs can be efficiently internalized in a time-dependent manner, and the uptake efficiency reaches 70.8 % after incubation for 24 hours **(Figure 3B).**

Given the central role of oxidative stress in inflammatory activation, we next evaluated whether internalized CF-EVs could attenuate intracellular ROS accumulation in H_2_O_2_-induced oxidative stress models. Total intracellular ROS levels were quantified using the DCFH-DA probe combined with flow cytometry. CF-EVs markedly reduced ROS accumulation in a concentration-dependent manner, resulting in an approximately 10 %-15 % reduction in fluorescence intensity compared with the H_2_O_2_-treated model group **(Figures 3C-D)**. To exclude potential interference from soluble contaminants, an equivalent volume of vesicle-isolation supernatant was used as a control. No significant reduction in ROS fluorescence was observed in the supernatant-treated group, whereas both low- and high-dose CF-EVs significantly suppressed intracellular ROS levels** (Figure 3D)**, confirming that the antioxidant effect primarily originated from the vesicles themselves. Consistently, DHE staining further demonstrated that CF-EVs effectively scavenged H_2_O_2_-induced superoxide anions **(Figures 3E-F)**. Fluorescence imaging using DCFH-DA and DHE probes further provides intuitive evidence that CF-EVs can significantly inhibit the accumulation of total reactive oxygen and superoxide in cells **(Figures 3G-H).**

Subsequently, the anti-inflammatory activity of CF-EVs was evaluated in the RAW 264.7 inflammatory model stimulated by LPS. LPS stimulation significantly upregulated the expression levels of pro-inflammatory cytokines IL-6, IL-1β, and TNF-α, while CF-EVs treatment significantly inhibited their expression levels** (Figures 3I-K)**. Overall, the above results show that CF-EVs exert strong antioxidant and anti-inflammatory effects in macrophages by directly removing free radicals, reducing intracellular oxidative stress, and inhibiting the expression of pro-inflammatory cytokines, thus providing mechanism-level support for their therapeutic potential in UC.

### CF-EVs promote the restoration of intestinal epithelial barrier function *in vitro*

To investigate the direct protective effects of CF-EVs on intestinal epithelial barrier function, a series of experiments was conducted using the human colorectal adenocarcinoma Caco-2 cell line. First, the cellular uptake of CF-EVs by Caco-2 cells was evaluated. Dil-labeled CF-EVs were co-incubated with Caco-2 monolayers for different time periods, followed by fluorescence microscopy observation and quantitative flow cytometric analysis. The results show that CF-EVs have time-dependent ingestion behavior. The intake efficiency reaches about 43.3 % at 12 h, and tends to the plateau phase at 24 h (44.9 %)** (Figures 4A-B)**, indicating that intestinal epithelial cells can efficiently internalize CF-EVs.

Subsequently, an *in vitro* Caco-2 single-layer intestinal epithelial barrier model was established (see **Figure 4C** for the diagram), and the integrity of the barrier was evaluated by real-time monitoring of cross-epithelial resistance (TEER). H_2_O_2_ and TNF-α combined treatment to simulate inflammatory intestinal barrier injury. As shown in **Figure 4D**, H_2_O_2_ + TNF-α stimulation significantly reduces the TEER value of the Caco-2 single layer, while CF-EVs pretreatment can significantly reduce the decline of TEER, indicating that CF-EVs have an obvious protective effect on the integrity of the epithelial barrier.

To further elucidate the protective mechanism of CF-EVs on the intestinal epithelial barrier, the expression of tight junction proteins and inflammatory responses was subsequently examined. Immunofluorescence staining revealed that, compared with the model group, the fluorescence intensities of the tight junction proteins ZO-1 and Occludin were markedly enhanced in CF-EVs-treated Caco-2 cells **(Figures 4E and G)**. Quantitative analysis further confirmed this trend** (Figures 4F and H)**, indicating that CF-EVs restored the expression and membrane localization of the tight junction proteins ZO-1 and Occludin.

The transcriptional levels of tight junction- and inflammation-related genes were further assessed by RT-qPCR. The results showed that pretreatment with CF-EVs significantly upregulated the mRNA expression levels of ZO-1 and Occludin **(Figures 4I-J)**. In contrast, CF-EVs treatment significantly attenuated the H_2_O_2_ + TNF-α-induced transcriptional elevation of the pro-inflammatory cytokines IL-6, IL-1β, and TNF-α. **(Figures 4K-M)**. These findings were further confirmed at the transcriptional level that CF-EVs not only enhanced the expression of barrier-associated structural proteins but also inhibited inflammatory cytokine expression, thereby facilitating the repair of injured intestinal epithelial cells under inflammatory conditions. Taken together, these results demonstrate that CF-EVs effectively restore impaired intestinal epithelial barrier function through a dual mechanism involving the upregulation of tight junction proteins and suppression of inflammatory cytokine expression.

### Preparation and characterization of the CF-EVs@Inulin

CF-EVs isolated by differential centrifugation were resuspended in precooled inulin solution and incubated at 4 °C overnight to construct the CF-EVs@Inulin hydrogel. Scanning electron Microscope (SEM) imaging revealed that both blank and CF-EVs-loaded hydrogels exhibited a characteristic three-dimensional porous network architecture, with no obvious structural alterations following vesicle incorporation** (Figure 5A)**. Notably, the CF-EVs@Inulin hydrogel displayed excellent injectability and could be readily extruded through a 27G needle (0.4 mm inner diameter), supporting its suitability for oral gavage administration **(Figure 5A)**.

Encapsulation within the inulin hydrogel did not compromise the intrinsic antioxidant activity of CF-EVs. As evaluated by ABTS•⁺, •OH, DPPH•, and PTIO radical-scavenging assays, CF-EVs@Inulin retained the potent antioxidant capacity of free CF-EVs and exhibited stronger radical-scavenging activity than either CF-EVs or the inulin hydrogel alone, suggesting a synergistic antioxidant effect between the encapsulated vesicles and the hydrogel matrix** (Figures S2A-D)**.

The release behavior of CF-EVs was subsequently investigated by encapsulating Dil-labeled vesicles within the hydrogel and incubating them in buffers that mimic different gastrointestinal pH conditions (pH 1.5, 6.8, 7.4, and 8.8). Quantification of Dil fluorescence in the collected supernatants revealed a sustained, time-dependent release profile under all tested conditions, with approximately 50 % of CF-EVs released within 6 h and nearly 95 % released after 48 h** (Figures 5B-F)**. To further evaluate its stability during gastrointestinal transit, the degradation behavior of CF-EVs@Inulin was examined under acidic conditions. Although hydrogel degradation proceeded more rapidly at lower pH, the degradation rate remained below 5 % after 6 h and below 35 % after 12 h **(Figure 5G)**, indicating good resistance to gastric acid. Considering that gastric emptying generally occurs within 3-4 h, most of the encapsulated CF-EVs are expected to remain protected before reaching the intestine. Moreover, because inulin is resistant to digestion in the upper gastrointestinal tract but can be selectively fermented by colonic microbiota, the hydrogel is expected to further facilitate colon-targeted release of CF-EVs 45.

Rheological analysis showed that the storage modulus (G′) consistently exceeded the loss modulus (G″) for all hydrogel formulations, confirming the formation of a predominantly elastic gel network. Incorporation of CF-EVs further increased the G′ of the inulin hydrogel, which may result from two factors. First, hydrogen-bonding interactions between membrane phospholipids and proteins on the surface of CF-EVs and the inulin network may strengthen intermolecular interactions. Second, the nanosized vesicles may occupy the pores within the three-dimensional hydrogel network, leading to a denser microstructure with an increased degree of crosslinking and enhanced mechanical stability **(Figure 5H)**. Meanwhile, the characteristic shear-thinning behavior of the inulin hydrogel was well preserved after CF-EV incorporation, indicating that vesicle encapsulation had no adverse effect on its rheological properties **(Figure 5I)**.

The gastrointestinal stability of the delivery system was further assessed by sequential exposure to simulated gastric fluid (SGF) and simulated intestinal fluid (SIF). SEM images showed that although simulated digestion caused slight disruption of the hydrogel microstructure, its three-dimensional porous architecture remained largely intact **(Figures S3A-B)**. Characterization of the released vesicles revealed that the average particle size decreased from 487.5 nm to 290.0 nm, accompanied by a marked reduction in the PDI from 0.717 to 0.212 **(Figures S3C-D)**, indicating that CF-EVs were released predominantly as uniformly dispersed vesicles rather than as aggregates. Taken together, these findings demonstrate that CF-EVs@Inulin combines sustained-release behavior with excellent gastrointestinal stability, enabling effective protection and controlled release of CF-EVs during gastrointestinal transit and providing strong support for its application as an oral therapeutic platform for ulcerative colitis.

### Biocompatibility evaluation of CF-EVs and CF-EVs@Inulin

In order to evaluate the biosafety of CF-EVs, RAW 264.7 and Caco-2 cells were exposed to CF-EVs with increasing concentrations, followed by CCK-8 activity analysis. As shown in **Figures S4A and B**, CF-EVs showed negligible cytotoxicity to both cells within the concentration range tested. Even at a concentration of 100 μg/mL, the vitality of RAW 264.7 cells remained at 99.6%, and that of Caco-2 cells remained at 91.36 %, indicating that they have excellent cell compatibility.

The blood compatibility of CF-EVs and CF-EVs@Inulin was further evaluated by* an in vitro* hemolysis experiment. Under the test conditions, neither of the two preparations caused obvious hemolysis **(Figure S4C)**, indicating that their red blood cell toxicity was extremely low and had good blood compatibility. Subsequently, the biosafety *in vivo* was evaluated in C57BL/6 mice, and CF-EVs or CF-EVs@Inulin (5 mg/kg) were administered continuously for 7 days. During the whole treatment period, the weight gain of the mice was normal, comparable to that of the control group treated with PBS, and there was no abnormality in behavior or appearance **(Figure S4D)**. Histopathological examination of major organs showed that there was no obvious tissue damage or structural abnormalities in both groups **(Figure S4E)**.

Consistently, hematological analysis showed that the main blood indicators, including lymphocytes, mononuclear cells, neutrophils, red blood cells and the total number of white blood cells, remained within the normal physiological range, and there was no significant change compared with the control group **(Figure S4F)**. Serum biochemical analysis further confirmed that there was no systemic toxicity, and there was no significant difference in liver function indicators (ALT, AST and TP) and renal function markers (UREA and CRE) between groups **(Figure S4G)**. These results show that CF-EVs and CF-EVs@Inulin have excellent biocompatibility and *in vivo* biosafety, supporting their application potential in oral treatment of ulcerative colitis.

### Biodistribution of oral CF-EVs@Inulin

To further evaluate the sustained release and colonic retention characteristics of CF-EVs@Inulin, DiR-labeled CF-EVs or CF-EVs@Inulin were given to healthy C57BL/6 mice orally, and their biological distribution was dynamically monitored by the living imaging system (IVIS). The fluorescence signal of the two preparations is mainly limited to the gastrointestinal tract, and no obvious off-target accumulation has been detected in the main organs **(Figures S5A-B)**, indicating that there are good gastrointestinal localization characteristics after oral administration.

Semi-quantitative analysis demonstrated that both groups reached maximal colonic fluorescence approximately 1 h after gavage; however, their clearance kinetics differed substantially. Free CF-EVs were rapidly eliminated, with fluorescence signals markedly declining after 6 h and becoming nearly undetectable by 24 h. In contrast, CF-EVs@Inulin exhibited significantly prolonged fluorescence retention within the colon, with strong residual signals still observable at 24 h **(Figure S5C)**. *Ex vivo* imaging of isolated colonic tissues further confirmed the enhanced retention capacity of the hydrogel formulation** (Figure S5D)**. Collectively, these findings demonstrate that inulin hydrogel encapsulation markedly prolongs the colonic residence time of CF-EVs and confers an effective colon-targeted sustained-release profile, thereby providing a favorable biodistribution basis for improving therapeutic efficacy against colitis.

### CF-EVs@Inulin attenuates DSS-induced acute colitis

To evaluate the therapeutic efficacy of CF-EVs@Inulin against colonic injury, a dextran sulfate sodium (DSS)-induced acute colitis model was established. Given the microbiota-dependent prebiotic activity of inulin and the proposed role of the gut microbiota in gut-kidney interactions, an antibiotic-mediated microbiota depletion experiment was additionally performed 46. Mice were pretreated with a broad-spectrum antibiotic cocktail containing ampicillin sodium (200 mg/kg), neomycin sulfate (200 mg/kg), metronidazole (200 mg/kg), and vancomycin hydrochloride (100 mg/kg) before DSS induction, and were randomly assigned to six groups: Control, DSS, DSS + Inulin, DSS + CF-EVs, DSS + CF-EVs@Inulin, and DSS + ABx + CF-EVs@Inulin** (Figure 6A)**.

During the experimental period, DSS treatment led to reduced survival and body weight, accompanied by increased disease activity index (DAI) scores. Treatment with Inulin, CF-EVs, or CF-EVs@Inulin significantly improved survival and alleviated DSS-induced body weight reduction and DAI elevation, among which CF-EVs@Inulin exhibited the most pronounced therapeutic effect **(Figures 6B-D)**. In contrast, depletion of the gut microbiota largely abolished these protective effects, as mice in the DSS + ABx + CF-EVs@Inulin group displayed survival rates, body weight loss, and DAI scores comparable to those of the DSS model group.

Exposure to DSS also leads to significant shortening of the colon, accompanied by severe mucosal damage characterized by diffuse congestion, edema, erosion, and ulcers. After CF-EVs@Inulin treatment, these pathological changes were significantly reduced, while there was no significant improvement in the mice in the antibiotic treatment group **(Figures 6E-G)**. Histological analysis further shows that CF-EVs@Inulin can effectively reduce epithelial destruction, crypt destruction (red arrows), and inflammatory cell infiltration (blue arrows) in the colon mucosa and submucosal layer **(Figure 6H)**.

In view of the key role of inflammatory cytokines in intestinal barrier dysfunction, the transcription levels of IL-1β, TNF-α, and IL-6 in colon tissue were quantitatively detected. DSS stimulation significantly upregulates the expression of all three cytokines, and Inulin, CF-EVs, or CF-EVs@Inulin treatment can significantly inhibit their expression, among which CF-EVs@Inulin has the strongest inhibitory effect. It is worth noting that this anti-inflammatory effect almost completely disappears after the microbiota is exhausted **(Figures 6I-K)**. Overall, the above results show that oral CF-EVs@Inulin can effectively improve DSS-induced acute colitis, and the treatment effect is better than that of Inulin or CF-EVs alone. Importantly, the protective effect after antibiotic treatment is almost completely lost, indicating that the beneficial effect of CF-EVs@Inulin is highly dependent on the complete intestinal microbiota, highlighting the synergistic mechanism involving microbiome reshaping and intestinal barrier repair.

### CF-EVs@Inulin restores intestinal barrier function

The integrity of the intestinal barrier is essential for maintaining gut-organ axis homeostasis. Therefore, the recovery of barrier function may re-establish physiological gut-kidney communication and reduce inflammation-related renal stress. In the DSS-induced acute colitis model, Alcian blue staining showed that DSS treatment significantly reduced the thickness of the mucus layer and the density of goblet cells, while CF-EVs@Inulin can effectively restore mucoprotein secretion and goblet cell abundance, indicating that the mucous barrier has been significantly repaired **(Figure 7A)**.

The expression and distribution of closely connected proteins were further detected by immunofluorescence staining. DSS exposure causes ZO-1 and Occludin fluorescent signals to be destroyed and discontinuous, reflecting the serious damage to the integrity of the tight connection of the epithelium **(Figure 7B)**. In contrast, CF-EVs@Inulin treatment significantly restored the expression of the two proteins and their continuous localization along the epithelial membrane, which was better than the Inulin or CF-EVs single group. Quantitative fluorescence analysis further confirmed the enhancement of ZO-1 and Occludin expression after CF-EVs@Inulin administration **(Figures 7C-D)**. It is worth noting that after the exhaustion of antibiotic-mediated microbiota, these recovery effects almost completely disappeared, and the fluorescence intensity of the ABx + CF-EVs@Inulin group was comparable to that of the DSS group. Consistently, RT-qPCR analysis shows that CF-EVs@Inulin can significantly improve the mRNA expression level of Occludin, ZO-1 and Claudin-1, and the effect is stronger than any single drug treatment **(Figures 7E-G)**. In contrast, microbiome depletion largely eliminated the improvement of these transcription levels, and no significant differences were observed between the ABx treatment group and the DSS group. Overall, these results show that CF-EVs@Inulin effectively restores the integrity of the intestinal barrier by co-repairing the mucus layer and the tight connection structure, and this protective effect is strongly dependent on the complete intestinal microbiota.

### Oral administration of CF-EVs@Inulin reverses DSS-induced gut microbiota dysbiosis

To investigate the effects of CF-EVs@Inulin on DSS-induced gut microbiota dysbiosis, fecal samples were subjected to 16S rRNA gene sequencing. β-diversity analyses consistently revealed profound alterations in microbial community architecture across groups. Principal component analysis (PCA) based on Bray-Curtis distances **(Figure 8A)**, principal coordinates analysis (PCoA, **Figure 8B**), and non-metric multidimensional scaling (NMDS) using Bray-Curtis, abundance Jaccard, and weighted UniFrac metrics **(Figures 8C-E)** all demonstrated a clear segregation between the DSS and Control groups. In contrast, samples from the Inulin, CF-EVs, and CF-EVs@Inulin groups clustered closer to the Control group, indicating partial restoration of microbial community structure. These intergroup differences were further validated by Kruskal-Wallis testing of β-diversity metrics **(Figure 8F)**.

Genus-level profiling of the top ten abundant taxa indicated that DSS exposure significantly increased potentially pathogenic Prevotellaceae while decreasing beneficial genera such as *Lactobacillus* and *Turicibacter* **(Figure 8G)**. Treatment with Inulin, CF-EVs, or CF-EVs@Inulin partially reversed these alterations, with CF-EVs@Inulin displaying the most pronounced recovery toward a control-like microbiota configuration. Notably, Inulin treatment specifically enriched *Ligilactobacillus*, suggesting a potential genus-level mediator of its protective effects. In contrast, antibiotic pretreatment (DSS + ABx + CF-EVs@Inulin) led to a collapse of commensal populations accompanied by overgrowth of *Escherichia-Shigella*, consistent with the loss of therapeutic efficacy in this group.

Boxplot-based comparisons of representative genera further confirmed that CF-EVs@Inulin significantly reduced Prevotellaceae abundance while enriching beneficial taxa, including *Bifidobacterium*, *Lactobacillus*, and *Turicibacter* **(Figures 8H-K)**. Heatmap visualization and top-taxa comparisons similarly indicated that the overall microbial configuration in the CF-EVs@Inulin group shifted markedly toward that of healthy controls **(Figures 8L-M)**.

Differential microbial biomarkers among the six groups were identified using linear discriminant analysis effect size (LEfSe) analysis and visualized by a genus-level cladogram **(Figure 8N)**. The DSS group (G2) exhibited a distinct microbial signature characterized by enrichment of several opportunistic and inflammation-associated bacterial genera, including Escherichia-Shigella, reflecting the severe disruption of intestinal microbial homeostasis during colitis development. Compared with the DSS group, mice treated with inulin alone (G3) or free CF-EVs (G4) showed partially shifted microbial profiles, with enrichment of specific commensal-associated genera, suggesting a moderate restoration of the intestinal microbial community.Notably, the CF-EVs@Inulin group (G5) displayed a distinct genus-level microbial pattern that was more closely associated with the healthy control group (G1), indicating enhanced remodeling of the dysbiotic microbiota induced by DSS. Several beneficial or commensal-associated genera, including Lactobacillus, Bifidobacterium, and members of Lachnospiraceae-related taxa, were differentially represented in the treated groups, suggesting that CF-EVs@Inulin may facilitate the recovery of a balanced microbial ecosystem. In contrast, antibiotic-treated mice receiving CF-EVs@Inulin (G6) exhibited a markedly altered microbial signature compared with the non-antibiotic treatment groups, reflecting profound ecological restructuring caused by microbiota depletion. Overall, genus-level LEfSe analysis demonstrated that CF-EVs@Inulin treatment promoted a microbial profile shift toward that of healthy controls, supporting its role in restoring intestinal microbial homeostasis during colitis.

Collectively, these findings demonstrate that oral CF-EVs@Inulin effectively reverses DSS-induced gut microbiota dysbiosis by promoting beneficial taxa while suppressing pathogenic populations. Mechanistically, the inulin hydrogel serves not only as a sustained-release carrier for CF-EVs in the colon but also as a fermentable substrate for gut microbiota, thereby enabling synergistic remodeling of microbial composition and restoration of intestinal microbial homeostasis.

### CF-EVs@Inulin alleviates colitis-associated early renal stress by restoring intestinal barrier integrity

Building on our previous findings that CF-EVs@Inulin effectively restores DSS-induced intestinal barrier integrity, we hypothesized that it may also attenuate early renal stress by limiting the systemic translocation of gut-derived toxins, particularly LPS, under conditions of increased intestinal permeability. To test this, the impact of orally administered CF-EVs@Inulin on the gut-kidney axis was systematically evaluated in a DSS-induced acute colitis model.

Intestinal permeability was initially evaluated using the FITC-dextran assay. As shown in **Figure 9A**, DSS treatment led to a marked elevation in serum FITC fluorescence, indicating severe disruption of the intestinal barrier. In contrast, administration of CF-EVs@Inulin substantially reduced FITC leakage, suggesting effective restoration of intestinal integrity. Consistently, the level of LPS in the serum and kidneys of DSS mice increased significantly** (Figures 9B-C)**, suggesting substantial translocation of luminal endotoxin into the circulation and kidney, where it likely contributes to early renal stress.

To elucidate the renal response to LPS challenge, we assessed the activation of the TLR4/NF-κB signaling cascade. As depicted in the results, DSS administration markedly upregulated TLR4 expression in kidney tissues and concurrently enhanced the phosphorylation of NF-κB p65, as reflected by a significant elevation in the p-p65/p65 ratio. In contrast, CF-EVs@Inulin significantly inhibited TLR4 expression and p65 phosphorylation **(Figure 9D and Figures S6A-C)**, indicating that it effectively inhibited the LPS-TLR4-NF-κB signaling pathway. Accordingly, after CF-EVs@Inulin treatment, the biochemical indicators of serum renal function, including uric acid (UA), creatinine (CRE), and urea (UREA), were significantly improved, and the therapeutic effect was better than that of Inulin or CF-EVs alone **(Figures 9E-G)**.

Histopathological analysis further shows that DSS induces early kidney injury, manifested as inflammatory infiltration, focal hemorrhagic lesions **(Figures 9H-I),** and abnormal glycogen accumulation in renal tubular epithelial cells **(Figure 9J)**. CF-EVs@Inulin significantly alleviated these changes, while mice in the antibiotic treatment group (DSS + ABx + CF-EVs@Inulin) did not see a significant improvement. At the molecular level, RT-qPCR analysis showed that DSS significantly upregulated the kidney injury markers KIM-1 and NGAL, confirming early renal tubular stress** (Figures 9K-L)**. CF-EVs@Inulin significantly inhibited their expression, which was more effective than single-drug treatment, and this protective effect disappeared in the ABx group. At the same time, CF-EVs@Inulin treatment also significantly reduced the anti-inflammatory mediators CCL-2 and ICAM-1** (Figures 9M-N)**, further supporting its anti-inflammatory effect in the kidneys.

Spearman correlation analysis showed that serum LPS levels were strongly positively correlated with kidney injury markers KIM-1 and NGAL (R > 0.89, *p* < 0.0001)** (Figures 9O-P)**. Correlation heat map analysis further reveals the close relationship between intestinal permeability (FITC-dextran), circulating LPS, and multiple renal dysfunction indicators (including KIM-1, NGAL, UA, CRE, and UREA)** (Figure 9Q)**, which jointly supports barrier destruction and kidney stress. Connected causal gut-renal axis.

In summary, CF-EVs@Inulin inhibits kidney TLR4/NF-κB activation by restoring the integrity of the intestinal barrier and reducing systemic LPS displacement, thus reducing the activation of colitis-related early renal stress. It is worth noting that this protective effect depends on the intestinal microbiota, highlighting the core role of the intestinal-renal axis in the progression of the disease.

## Discussion

In this study, we successfully isolated CF-EVs for the first time and developed an oral prebiotic delivery system, CF-EVs@Inulin, based on an inulin hydrogel. We demonstrate that this platform effectively alleviates DSS-induced colitis and associated early renal stress by restoring intestinal barrier integrity, reshaping gut microbiota composition, and preventing LPS translocation.

CF-EVs displayed a characteristic cup-shaped morphology with an average diameter of ~157 nm and exhibited potent broad-spectrum activity. Plant-derived extracellular vesicles are known to carry a variety of functional biomolecules, including membrane-associated proteins, lipids, and secondary metabolites 47, 48. Given that *Cortex Fraxini* is enriched in phenolic compounds such as coumarins and flavonoids 49, Consistent with the chemical characteristics of *Cortex Fraxini*, LC-MS/MS analysis further revealed that CF-EVs carry several antioxidant-related phytochemicals, particularly coumarin derivatives such as* esculetin* and* fraxetin*. These antioxidant phytochemicals may be partially encapsulated or associated with CF-EVs, thereby contributing to their radical-scavenging activity. Although multiple complementary characterization methods together with EV-depleted supernatant controls support that the observed biological activities were primarily associated with the vesicular fraction, the absence of universally accepted molecular markers for plant-derived extracellular vesicles remains a limitation of the current field. Future studies should focus on establishing standardized purification protocols and identifying reliable molecular markers to further strengthen plant EVs characterization. These antioxidant properties are consistent with the coumarin- and polyphenol-rich composition of *Cortex Fraxini*. It is worth noting that CF-EVs can be efficiently internalized by macrophages and intestinal epithelial cells, reduce ROS accumulation in cells, inhibit the expression of NF-κB-mediated pro-inflammatory cytokines (IL-6, TNF-α, and IL-1β), and upregulate the tightly connected proteins ZO-1 and Occludin, so as to promote the repair of the epithelial barrier outside the body.

In order to achieve colon-targeted delivery and overcome gastrointestinal degradation, CF-EVs were encapsulated in inulin-based hydrogels through cooling-induced gelation strategies. The obtained hydrogel showed excellent gastrointestinal stability. Under simulated digestion conditions, the degradation rate was less than 5 % within 6 hours, and the cumulative release rate of CF-EVs within 48 hours was about 95 %. *In vitro* imaging further confirmed that compared with free CF-EVs, CF-EVs@Inulin has a longer colonic residence time 24 hours after administration, highlighting its sustained release and colon targeting ability.

In the colitis model, CF-EVs@Inulin significantly improved the survival rate, reduced weight loss, reduced colon shortening, and alleviated histopathological damage. The therapeutic effect was better than that of Inulin or CF-EVs alone. Importantly, after the intestinal microbiome mediated by broad-spectrum antibiotics is exhausted, these protective effects basically disappear, indicating that they are highly dependent on microbial homeostasis. Consistently, 16S rRNA sequencing shows that CF-EVs@Inulin reverses the DSS-induced flora imbalance by reducing the abundance of potential pathogenic bacteria such as Prevotellaceae, while enriching beneficial bacteria such as *Bifidobacterium* and* Lactobacillus*, thereby restoring a microbial configuration resembling that of healthy controls. These changes work together with the recovery of the mucus layer and the enhancement of close connection, which promotes the comprehensive repair of the intestinal barrier. Although antibiotic depletion experiments support the participation of intestinal microbiota in the therapeutic effect of CF-EVs@Inulin, broad-spectrum antibiotics may also affect host immune response and intestinal physiology independently of microbial depletion. Therefore, the current research results should be interpreted as evidence of the participation of microbiota, rather than a certain proof of causality 50. Future research on sterile mice or fecal microbial transplantation will further establish the causal contribution of intestinal microbiota to the therapeutic effect of CF-EVs@Inulin.

More importantly, we have clarified the gut-kidney axis mechanism behind the kidney protection of CF-EVs@Inulin. DSS-induced colitis increases intestinal permeability, resulting in increased serum and kidney LPS levels, activating the kidney TLR4/NF-κB (p-p65) signal axis, and upregulating the early kidney injury markers KIM-1 and NGAL, accompanied by impaired renal function. By restoring the integrity of the barrier, CF-EVs@Inulin significantly reduces systemic LPS translocation, thus inhibiting TLR4/NF-κB activation and reducing kidney inflammation and tissue damage. Spearman rank correlation analysis shows that there is a significant, strong positive correlation (R > 0.85) between circulating LPS and kidney injury markers, which emphasizes the core role of intestinal endotoxemia in driving kidney stress during colitis.

In summary, our research results show that CF-EVs@Inulin integrates the prebiotic function of inulin with the barrier protection and biological activity characteristics of CF-EVs to restore intestinal homeostasis and inhibit LPS-driven renal stress. This study provides a promising extracellular vesicle delivery strategy based on prebiotics for ulcerative colitis and its intestinal-renal axis-related extraintestinal complications. Although this study identifies LPS translocation as a contributing factor to the potential mechanism of gut-kidney communication, this mechanism may not be enough to fully explain the complex biological interactions involved. Future research will give priority to multi-omics analysis of CF-EVs, verification in chronic disease models, and deeply clarify the mechanism of specific microbial flora and metabolites behind the therapeutic effect.

## Conclusion

In this study, we successfully isolated extracellular vesicles from *Cortex Fraxini* and developed an oral prebiotic delivery system, CF-EVs@Inulin, using an inulin hydrogel. This platform combines colon-targeted sustained release, prebiotic activity, and the intrinsic antioxidant, anti-inflammatory, and barrier-repairing properties of natural vesicles. In the UC mouse model, CF-EVs@Inulin effectively restored the mucus layer, tight junction integrity, and gut microbiota homeostasis, with its therapeutic efficacy strongly dependent on an intact gut microbiota. More importantly, we demonstrated that CF-EVs@Inulin alleviated colitis-associated renal injury by interrupting the gut-kidney axis cascade of “intestinal barrier disruption → LPS translocation → renal TLR4/NF-κB activation → early renal stress.” These findings reveal the therapeutic potential of CF-EVs and provide a promising prebiotic-based EV delivery strategy for targeting intestinal barrier dysfunction and gut-kidney axis-related complications in UC.

## Methods and Materials

### Isolation and purification of CF-EVs

CF-EVs were isolated using differential ultracentrifugation combined with sucrose density gradient purification. Briefly, dried *Cortex Fraxini* bark was cut into small pieces (3-5 cm) and homogenized in precooled phosphate-buffered saline (PBS, pH 7.4) at a material-to-liquid ratio of 1: 5 (g/mL) using a Polytron homogenizer, followed by extraction at 4 °C for 12 h. The homogenate was filtered through a 220 μm mesh and subjected to sequential centrifugation at 500 × *g* (10 min), 1,000 × *g* (10 min), 2,000 × *g* (20 min), 3,000 ×* g* (30 min), 5,000 × *g* (30 min), and 10,000 × *g* (60 min) at 4 °C to remove cell debris and large particles. The resulting supernatant was ultracentrifuged at 130,000 × *g* for 90 min to obtain crude EV pellets. The pellets were resuspended in sterile PBS and further purified by sucrose density gradient ultracentrifugation (8 %, 30 %, 45 %, and 60 %) at 130,000 × *g* for 90 min using an Optima XE-90 ultracentrifuge (Beckman Coulter). The fraction located between the 30 % and 45 % sucrose layers was collected and subjected to a second ultracentrifugation step to obtain purified CF-EVs.

### Cell culture

RAW 264.7 murine macrophages and Caco-2 human intestinal epithelial cells were obtained from Zishan Biotechnology (China). RAW 264.7 cells were cultured in DMEM, while Caco-2 cells were maintained in MEM supplemented with 1 % non-essential amino acids (NEAA). Both media contained 10 % fetal bovine serum (FBS) and 1 % penicillin-streptomycin. Cells were cultured at 37°C in a humidified incubator with 5 % CO₂.

### TEER measurement

Caco-2 cells were cultured on Transwell inserts to establish an intestinal epithelial barrier model. TEER values were measured using a Millicell® ERS-3.0 voltohmmeter and calculated as: TEER (Ω·cm²) = (Rm - Ri) × A. Barrier formation was considered successful when TEER values exceeded 300 Ω·cm² 51. Cells were pretreated with CF-EVs (10 or 100 μg/mL) for 12 h, followed by stimulation with TNF-α (30 ng/mL, 12 h) and H_2_O_2_ (600 μM, 4 h) to induce barrier injury.

### Preparation and characterization of CF-EVs@Inulin

CF-EVs@Inulin hydrogel was prepared by dissolving 0.6 g inulin in 1 mL PBS under stirring, followed by heating at 80°C for 10 min (800 rpm). After cooling to room temperature, CF-EVs obtained by differential ultracentrifugation were resuspended in the inulin solution, vortexed, ultrasonicated, and incubated at room temperature for 12 h to form the hydrogel. Blank inulin hydrogel and Dil-labeled CF-EVs@Inulin hydrogel were prepared similarly. For SEM observation, hydrogels were swollen in water for 4 h, frozen in liquid nitrogen, freeze-dried, and characterized by SEM.

### *In vitro* degradation of CF-EVs@Inulin

CF-EVs@Inulin hydrogels were incubated at 37 °C in buffers of different pH values (1.5, 6.8, 7.4, and 8.8). At designated time points, the supernatant was removed and the remaining hydrogels were weighed. The degradation rate was calculated as^41^:



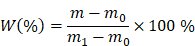



where m_0_ is the weight of the empty tube, m_1_ is the initial total weight of the tube and hydrogel, and m is the remaining weight at each time point.

### *In vitro* release of CF-EVs@Inulin

Dil-labeled CF-EVs@Inulin hydrogel (1 g) was incubated in PBS buffers at different pH values (1.5, 6.8, 7.4, and 8.8). At predetermined time points, supernatants were collected, and fluorescence intensity at 565 nm was measured to evaluate CF-EVs release profiles.

### Gastrointestinal stability of CF-EVs@Inulin

To evaluate gastrointestinal stability, CF-EVs@Inulin hydrogels were sequentially incubated in simulated gastric fluid (SGF, pH 1.5) at 37 °C for 120 min and simulated intestinal fluid (SIF, pH 6.8) for an additional 60 min. Samples were collected before and after digestion, and particle size distribution was analyzed by DLS.

### *In vivo* biodistribution of CF-EVs@Inulin

CF-EVs and CF-EVs@Inulin were labeled with the near-infrared dye DiR and orally administered to mice at 5 mg/kg. At designated time points within 24 h, whole-body fluorescence imaging was performed using the IVIS under isoflurane anesthesia. After sacrifice, major organs and the gastrointestinal tract were collected for *ex vivo* imaging. Fluorescence intensity was quantified to evaluate the biodistribution and colonic retention of CF-EVs and CF-EVs@Inulin.

### Animal model

Male C57BL/6 mice (6 weeks old, ~22 g) were acclimated for 7 days before experiments. Acute colitis was induced by administering 3 % DSS (36-50 kDa, MP Biomedicals) in drinking water for 7 days, followed by normal water. Mice were randomly divided into six groups (n = 6/group): Control, DSS, DSS + Inulin, DSS + CF-EVs, DSS + CF-EVs@Inulin, and DSS + Abx + CF-EVs@Inulin.

For gut microbiota depletion, mice in the Abx group received a cocktail of ampicillin, neomycin, metronidazole, and vancomycin by oral gavage for 3 consecutive days before treatment. From day 7, mice were orally administered Inulin, CF-EVs, or CF-EVs@Inulin (5 mg/kg in 100 μL PBS) once daily until the endpoint, while Control and DSS groups received equal volumes of PBS. Body weight and disease activity were monitored throughout the experiment. At the end of the experiment, all mice were euthanized by CO₂ inhalation followed by cervical dislocation. All animal experiments were performed following the Principles of Laboratory Animal Care and Guidelines of the Laboratory Animal Care Committee of Xi’an Jiaotong University (No: XJTUAE2024-1005).

### Intestinal permeability assay

Intestinal permeability was assessed using FITC-dextran (4 kDa). Mice were orally ligated with FITC- dextran (0.6 mg/g body weight), and blood samples were collected after 4 hours. Plasma fluorescence intensity (Ex/Em: 485/530 nm) is used as an indicator of intestinal permeability.

### Gut microbiota analysis

The original sequencing reading segment uses the QIIME2 platform with DADA2 for mass filtering, denoising, and amplified sub-sequence variant (ASV) generation. Subsequently, β-diversity, microbial composition, and differential abundance were analyzed.

### Histological analysis

The colon and kidney tissues were fixed, embedded and sliced for histological analysis. H&E, Alcian blue, and PAS staining were carried out, respectively, to evaluate tissue morphology, mucus secretion, epithelial integrity, and renal glycogen accumulation.

### Immunofluorescence analysis

Caco-2 cells and frozen colon sections were fixed, permeabilized, and blocked with 1 % BSA. Samples were incubated overnight at 4°C with primary antibodies against ZO-1 and Occludin, followed by fluorescent secondary antibodies and DAPI nuclear staining. Fluorescence images were collected by microscope and quantitatively analyzed using ImageJ software.

### Biocompatibility and biosafety evaluation

RAW 264.7 and Caco-2 cells are co-incubated with different concentrations of CF-EVs, and the cell vitality is evaluated by CCK-8 assay. For *in vivo* biosafety assessment, mice were given CF-EVs or CF-EVs@Inulin (5 mg/kg) every day for 7 days. Conduct hematology and serum biochemical analysis, and major organs were collected for H&E staining and histopathological evaluation.

### Statistical analysis

The data are expressed as the mean value ± standard deviation (SD) of three independent experiments. Student's t-test or single/two-factor analysis of variance (ANOVA) is used for statistical analysis, followed by an appropriate post-test. Differences were considered statistically significant at *p < 0.05, **p < 0.01, and ***p < 0.001; ns, not significant.

## Supplementary Material

Supplementary methods, figures and table.

## Figures and Tables

**Figure 1:**
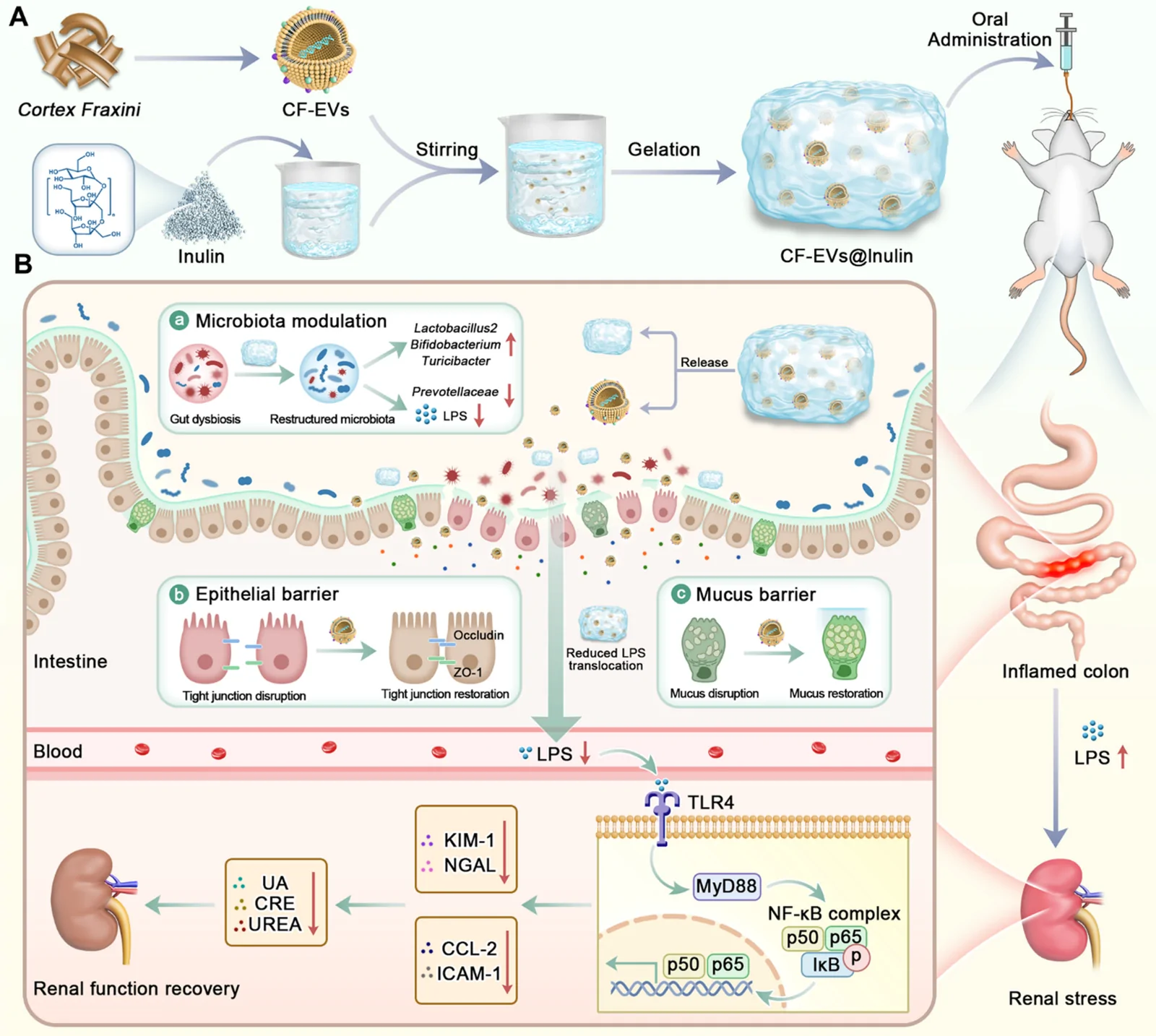
** Schematic illustration of the fabrication of CF-EVs@Inulin and its oral therapeutic effects against colitis and associated early renal stress. (A) CF-EVs were isolated from *Cortex Fraxini* and subsequently mixed with an inulin aqueous solution to construct the CF-EVs@Inulin hydrogel system. (B) Oral administration of CF-EVs@Inulin alleviated UC and associated early renal stress by restoring multiple** intestinal barrier functions.

**Figure 2:**
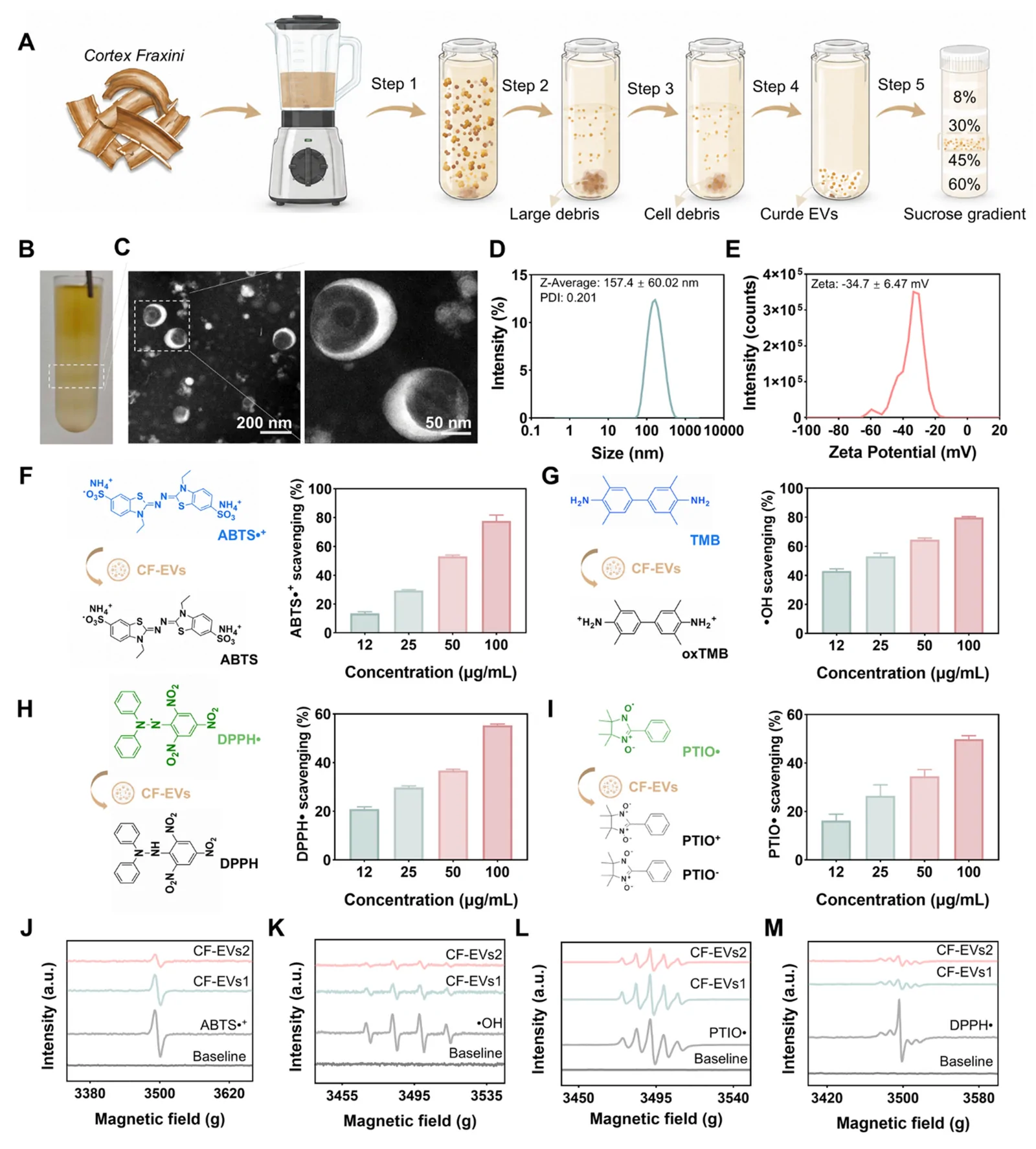
** Physicochemical characterization and antioxidant activity of CF-EVs.** (A) Schematic illustration of the extraction procedure for CF-EVs. (B) Sucrose density gradient (30-45 %) purification of CF-EVs. (C) TEM images of CF-EVs enriched in the 30 %-45 % sucrose density fractions. (D) Hydrodynamic particle size distribution measured by DLS. (E) Zeta potential analysis. (F-I) *In vitro* free radical scavenging activities and corresponding schematic illustrations: (F) ABTS⁺ radical scavenging activity, (G) •OH scavenging activity, (H) DPPH radical scavenging activity, and (I) PTIO radical scavenging activity. (J-M) EPR spectra and quantitative analyses of radical scavenging activity: (J) ABTS•⁺, (K) •OH, (L) DPPH•, (M) PTIO radicals. CF-EVs1 and CF-EVs2 represent concentrations of 10 μg/mL and 100 μg/mL, respectively.

**Figure 3:**
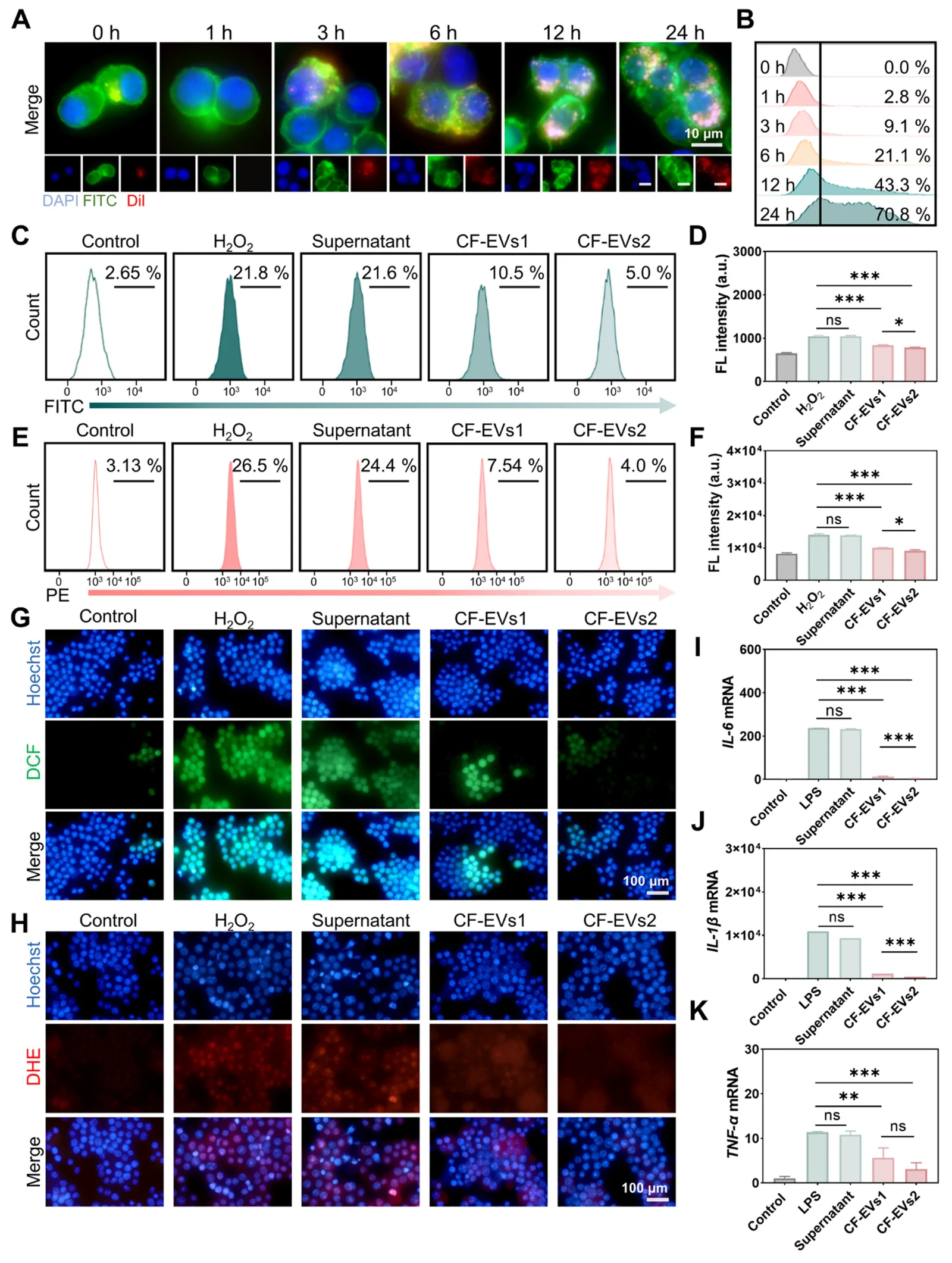
***In vitro* antioxidant and anti-inflammatory activities of CF-EVs.** (A) Cellular uptake of Dil-labeled CF-EVs by RAW 264.7 cells after 24 h of incubation. (B) Flow cytometric analysis of CF-EVs uptake at different time points. (C-D) Intracellular total ROS levels were detected by DCFH-DA staining and quantified by mean fluorescence intensity (MFI). Vesicle-isolation supernatant was used as a control. (E-F) Superoxide anion levels were detected by DHE staining and quantified by MFI. (G-H) Representative fluorescence microscopy images showing intracellular total ROS (DCF) and superoxide anion (DHE) levels. (I-K) Effects of CF-EVs on the mRNA expression of IL-6, IL-1β, and TNF-α in LPS-induced RAW 264.7 cells, as assessed by RT-qPCR. CF-EVs1 and CF-EVs2 represent CF-EVs at 10 and 100 μg/mL, respectively. Data are presented as mean ± SD (n = 3). *p < 0.05, **p < 0.01, ***p < 0.001; ns, not significant.

**Figure 4:**
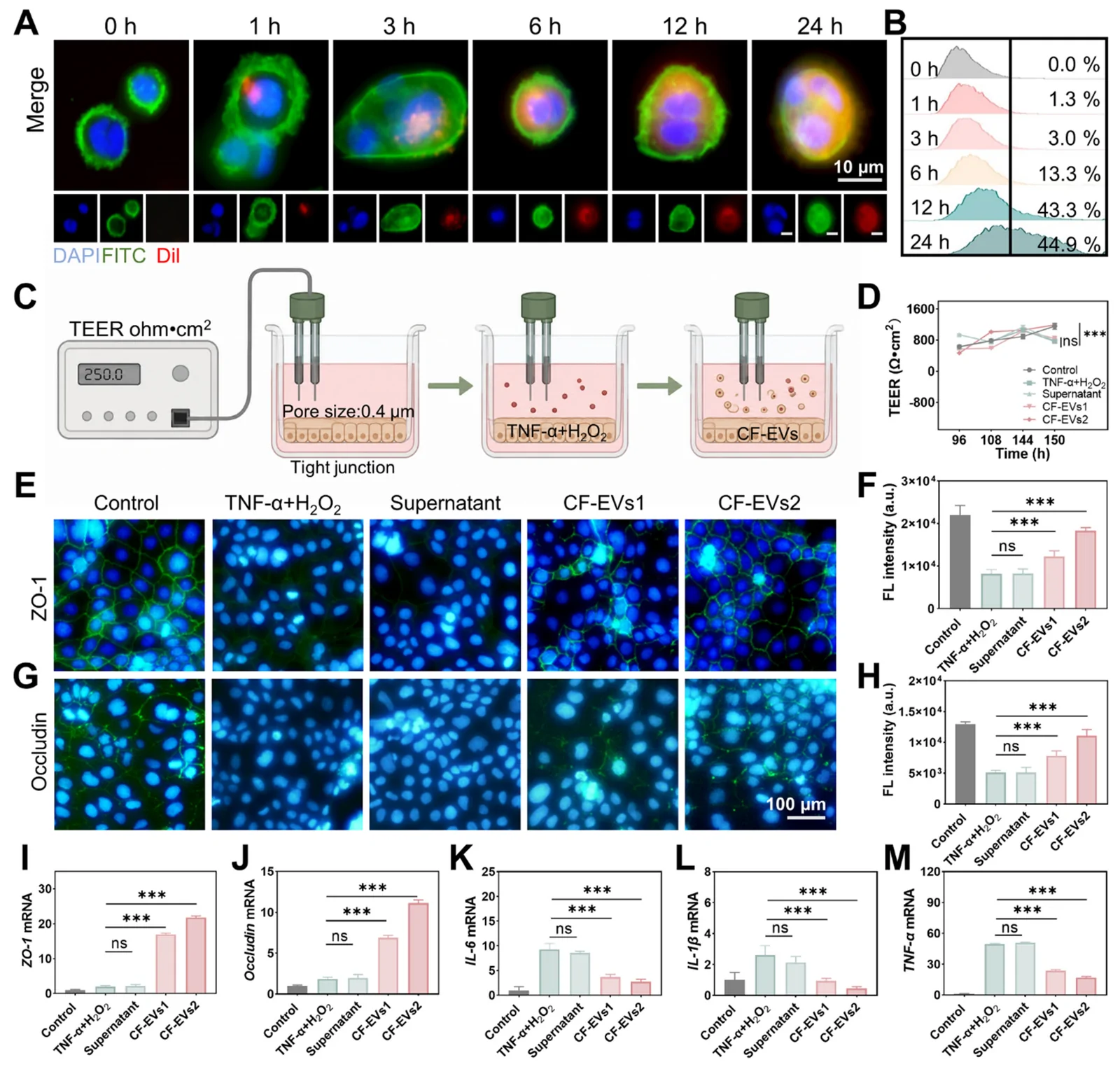
** CF-EVs mitigate TNF-α + H_2_O_2_-induced impairment of epithelial tight junction integrity.** Caco-2 cells were pretreated with CF-EVs for 12 h before co-stimulation with TNF-α and H_2_O_2_. CF-EVs1 and CF-EVs2 represent concentrations of 10 μg/mL and 100 μg/mL, respectively. (A) Uptake of Dil-CF-EVs (red) by Caco-2 cells; DAPI (blue) nuclear staining. (B) Quantitative flow cytometric analysis of the endocytic uptake efficiency of CF-EVs by Caco-2 cells. (C) Schematic illustration of the Caco-2 monolayer intestinal epithelial barrier model established using the Transwell system. (D) Real-time monitoring of TEER to evaluate barrier integrity (n = 3). (E, G) Representative immunofluorescence images of ZO-1 (E) and Occludin (G), with DAPI-stained nuclei (blue). (F-H) Quantitative analysis of the fluorescence intensities of ZO-1 (F) and Occludin (H). (I-J) Relative mRNA expression levels of ZO-1 (I) and Occludin (J) determined by RT-qPCR after 6 h of TNF-α + H_2_O_2_ stimulation (n = 3). (K-M) mRNA levels of IL-6 (K), IL-1β (L), and TNF-α (M) were measured by RT-qPCR. (L) (n = 3). Data are presented as mean ± SD (n = 3). **p < 0.01, ***p < 0.001; ns, not statistically significant.

**Figure 5:**
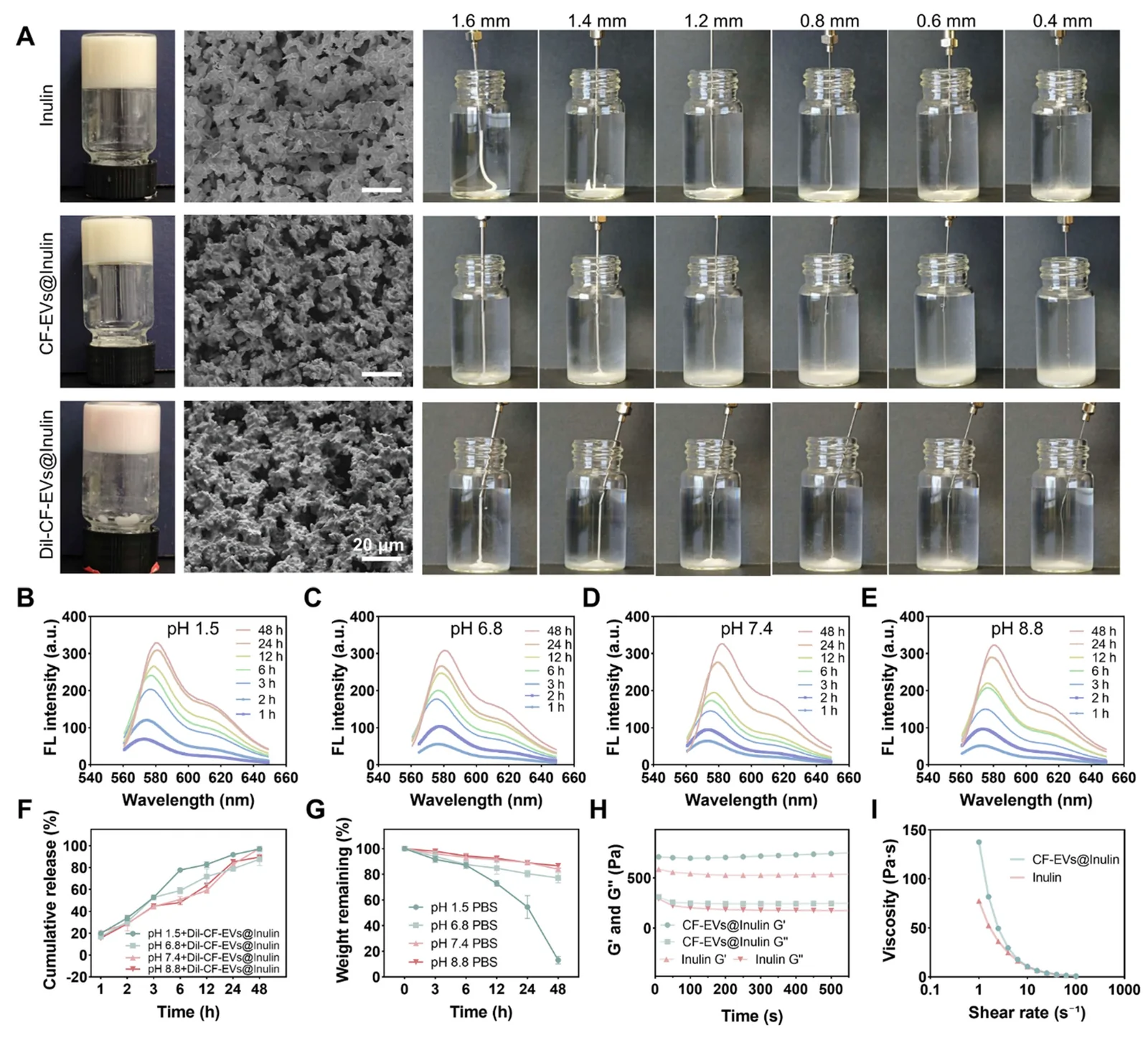
** Inulin hydrogel enables stable encapsulation and sustained release of CF-EVs.** (A) Representative photographs, SEM images, and injectability evaluation of inulin-based hydrogels, showing porous network structures and extrusion through different needle sizes. (B-F) The *in vitro* release curve of CF-EVs@Inulin at 37 °C and different pH buffers (1.5, 6.8, 7.4, and 8.8) is expressed by the cumulative fluorescence release of Dil-labeled CF-EVs. (G) CF-EVs@Inulin *in vitro* degradation curve under different pH conditions. (H) Storage modulus (G′) and loss modulus (G″) of Inulin and CF-EVs@Inulin determined by rheological analysis. (I) Viscosity curves of Inulin and CF-EVs@Inulin.

**Figure 6:**
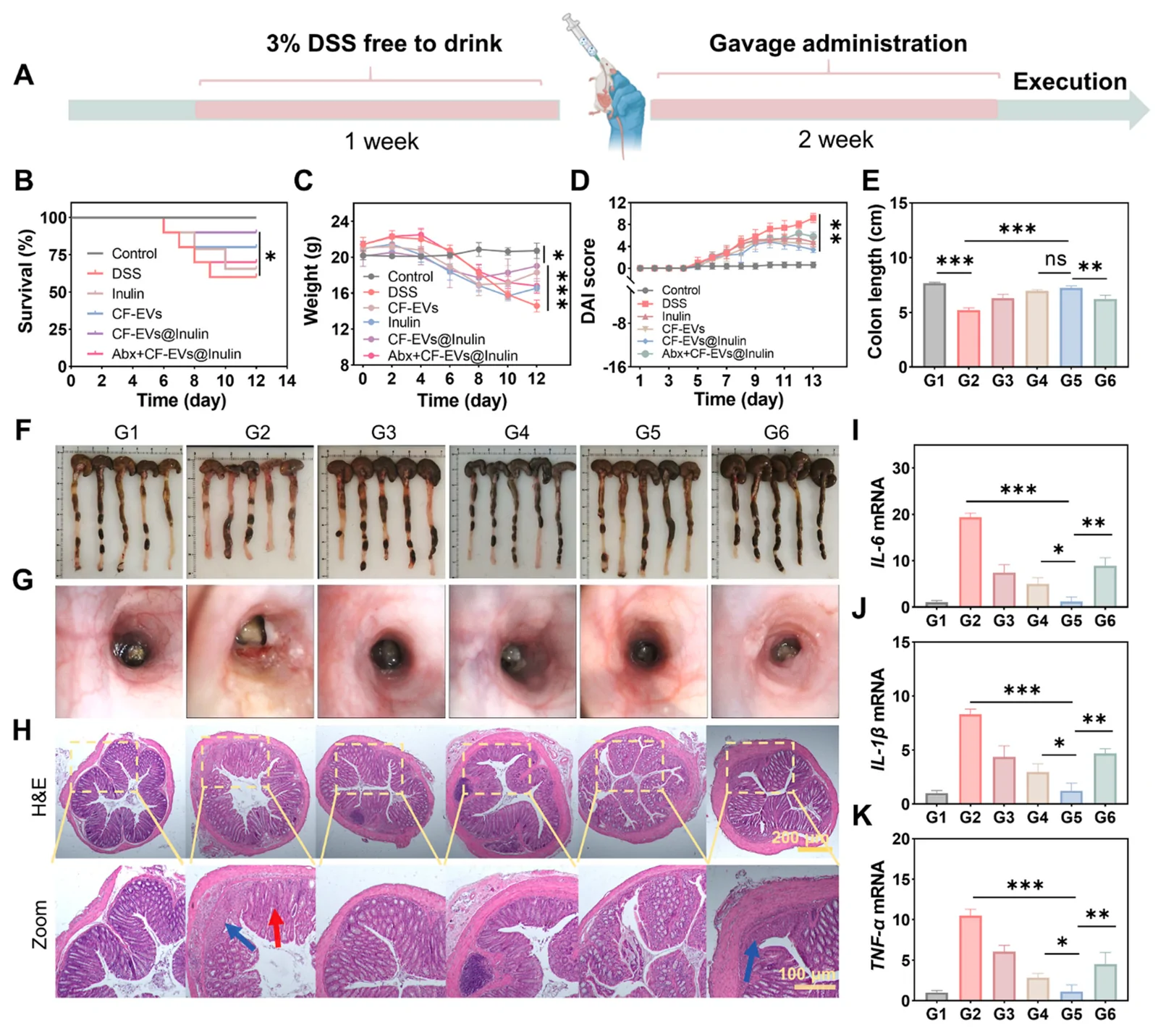
** Oral CF-EVs@Inulin can alleviate DSS-induced acute colitis.** (A) Experimental design and treatment schedule of DSS-induced colitis. (B) Survival curve (n = 6). (C) Weight change. (D) Disease Activity Index (DAI) score (n = 6). (E) Colon length measurement. (F-G) Representative images of colon morphology and colonoscopy. (H) H&E staining of colon tissue shows epithelial damage, crypt destruction (red arrow), and inflammatory cell infiltration (blue arrow). (I-K) mRNA expression levels of IL-6, IL-1β and TNF-α in colon tissue. G1, control group; G2, DSS group; G3, DSS + Inulin group; G4, DSS + CF-EVs group; G5, DSS + CF-EVs@Inulin group; G6, DSS + Abx + CF-EVs@Inulin group. Data are presented as mean ± SD (n = 3). *p < 0.05, **p < 0.01, ***p < 0.001; ns, not significant.

**Figure 7:**
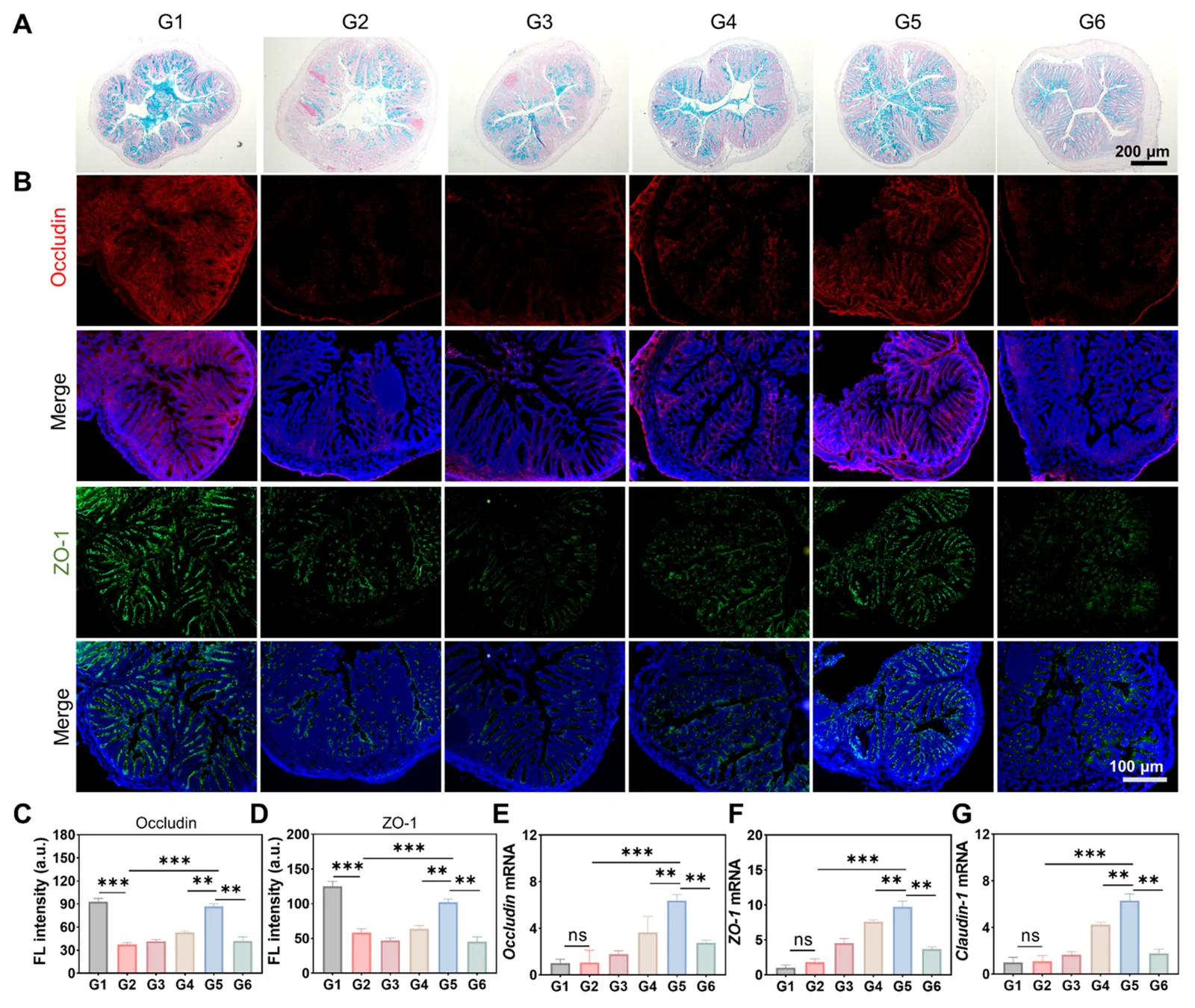
** CF-EVs@Inulin restores intestinal epithelial and mucus barrier function in DSS-induced acute colitis.** (A) Alcian Blue staining of colon sections showing the mucus layer and goblet cells. (B) Representative immunofluorescence images of Occludin and ZO-1 in colon tissues, with DAPI nuclear staining (blue). (C-D) Quantification of Occludin and ZO-1 fluorescence intensity (n = 3). (E-G) Colonic mRNA expression of Occludin, ZO-1, and Claudin-1 (n = 3). G1, Control; G2, DSS; G3, DSS + Inulin; G4, DSS + CF-EVs; G5, DSS + CF-EVs@Inulin; G6, DSS + Abx + CF-EVs@Inulin. Data are presented as mean ± SD (n = 3). **p < 0.01, ***p < 0.001; ns, not significant.

**Figure 8:**
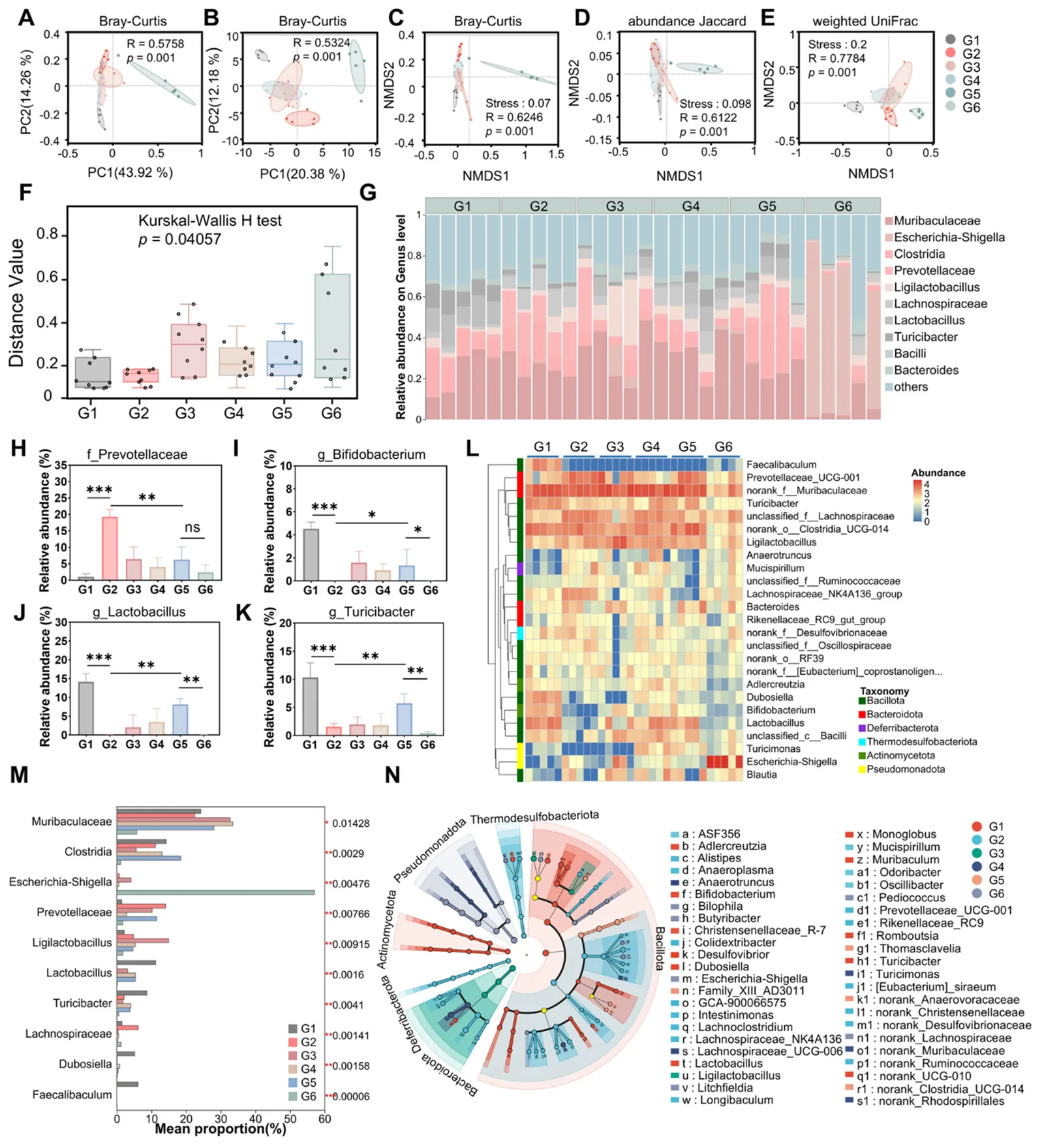
** Oral administration of CF-EVs@Inulin modulates gut microbiota composition.** G1, Control; G2, DSS; G3, DSS + Inulin; G4, DSS + CF-EVs; G5, DSS + CF-EVs@Inulin; G6, DSS + Abx + CF-EVs@Inulin. (A-E) β-diversity analysis of gut microbiota, including PCA, PCoA, and NMDS analyses using Bray-Curtis, Abund-Jaccard, and Weighted UniFrac distances. Ellipses indicate 95 % confidence intervals. (F) Rank-sum test of β-diversity differences among groups. (G) Stacked bar plot of the top 10 bacterial genera. (H-K) Relative abundance of representative bacterial taxa, including Prevotellaceae, *Bifidobacterium*, *Lactobacillus*, and *Turicibacter* (n = 3). (L) Heatmap of the top 25 bacterial taxa at the genus level. (M) Relative abundance of the top 10 differential genera among groups. (N) Differential microbial biomarkers among groups were identified using LEfSe analysis and visualized by genus-level cladogram. Data are presented as mean ± SD (n = 3). *p < 0.05, **p < 0.01, ***p < 0.001; ns, not significant.

**Figure 9:**
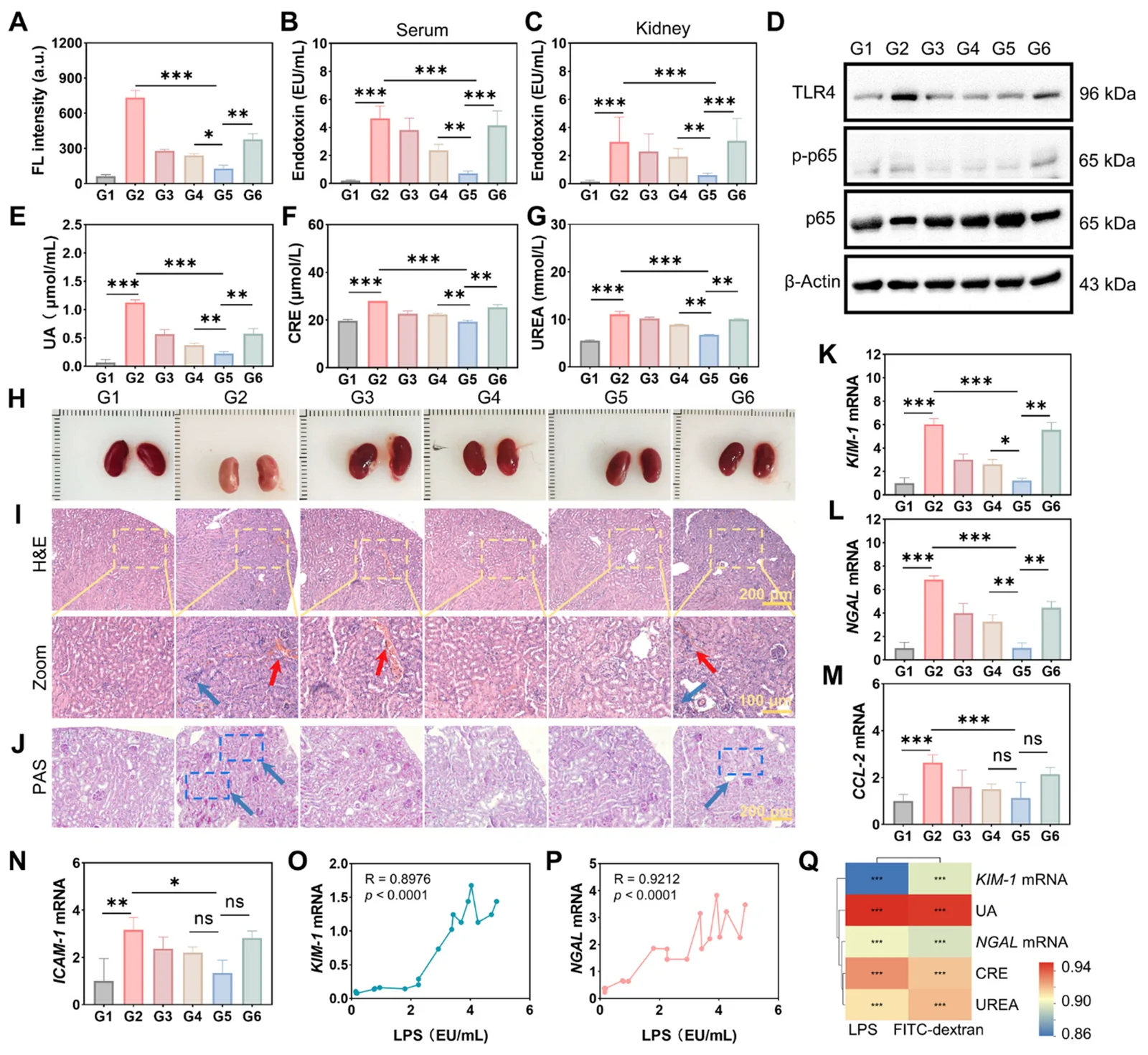
** Oral CF-EVs@Inulin alleviates DSS-induced colitis-associated early renal stress by restoring intestinal barrier integrity.** G1, Control; G2, DSS; G3, DSS + Inulin; G4, DSS + CF-EVs; G5, DSS + CF-EVs@Inulin; G6, DSS + Abx + CF-EVs@Inulin. (A) Serum FITC-dextran fluorescence intensity for intestinal permeability assessment (n = 3). (B, C) Serum and renal LPS levels. (D) Western blotting analysis of renal TLR4, p65, and p-p65 expression. (E-G) Serum renal function indicators, including UA, CRE, and UREA. (H) Representative gross morphology of kidneys. (I, J) Histological analysis of kidney tissues by H&E and PAS staining. Blue and red arrows indicate inflammatory cell infiltration and hemorrhagic lesions, respectively. (K-N) Renal mRNA expression of KIM-1, NGAL, CCL-2, and ICAM-1. (O, P) Spearman correlation analysis between serum LPS and renal injury markers KIM-1 and NGAL. (Q) Correlation heatmap among serum LPS, FITC-dextran, renal injury markers, and renal function indicators. Data are presented as mean ± SD (n = 3). *p < 0.05, **p < 0.01, ***p < 0.001; ns, not significant.

## Data Availability

Data supporting the findings of this study may be obtained from the corresponding author upon reasonable request.

## Abbreviations

IBD: inflammatory bowel disease
CF-EVs: Cortex Fraxini-derived extracellular vesicles
DSS: dextran sulfate sodium
CKD: chronic kidney disease
SCFAs: short-chain fatty acids
IS: indoxyl sulfate
pCS: p-cresyl sulfate
TMAO: trimethylamine N-oxide
RAS: renin-angiotensin system
CD: Crohn’s disease
UC: ulcerative colitis
TEM: Transmission electron microscopy
DLS: Dynamic light scattering
EPR: electron paramagnetic resonance
•OH: hydroxyl radical
ABTS•⁺: ABTS cation radicals
DPPH•: DPPH radicals
TEER: transepithelial electrical resistance
UA: uric acid
CRE: creatinine
