# Supplementary material for: Oral inulin prebiotic encapsulating Cortex Fraxini extracellular vesicles alleviates colitis and associated renal stress via reinforcing intestinal barriers and remodeling microbiota
Source: Theranostics. 2026 Sep 2;16(16):9093–112. doi: 10.7150/thno.139484 (PMC13684145; doi:10.7150/thno.139484)
Supplement: Supplementary file 1 [file thnov16p9093s1.pdf]

Oral inulin prebiotic encapsulating *Cortex Fraxini* extracellular vesicles alleviates colitis and associated renal stress via reinforcing intestinal barriers and remodeling microbiota

Meihong Chai[1#], Junlong Fu[2#], Mengjie Zhang[3], Xing Pei[4], Xingzhou Liu[2], Bing Xiao[1], Bowen Gao[2], Jingya Wei[2], Baosen Yue[1], Mingxin Zhang[5*], Mingzhen Zhang[5*], and Juan Jin[6*]

Affiliations

1. Department of Pharmacy, Xi'an Hospital of Traditional Chinese Medicine, Xi'an, Shaanxi, 710021, China
2. Department of Pharmacology, School of Basic Medical Sciences, Xi'an Jiaotong University, Xi'an, Shaanxi, 710061, China
3. School of Life Science; School of Interdisciplinary Science; Aerospace Center Hospital; Key Laboratory of Molecular Medicine and Biotherapy; Key Laboratory of Medical Molecule Science and Pharmaceutics Engineering; Beijing Institute of Technology, Beijing, 100081, China
4. Tianjin Key Laboratory of Food and Biotechnology, School of Biotechnology and Food Science, Tianjin University of Commerce, Tianjin, 300134, China
5. Department of Gastroenterology, The First Affiliated Hospital of Xi'an Medical University, Xi'an, Shaanxi, 710077, China
6. Department of Nephrology, the First Affiliated Hospital of Zhejiang Chinese Medical University (Zhejiang Provincial Hospital of Chinese Medicine), Hangzhou, Zhejiang, 310003, China

# The authors contribute equally to this work

∗ Authors for correspondence

Mingxin Zhang (ORCID: 0000-0002-3945-3387)
Email address: zmx3115@xiyi.edu.cn

Mingzhen Zhang (ORCID: 0000-0002-4686-6526)
Email address: mzhang21@xjtu.edu.cn

Juan Jin (ORCID: 0000-0003-2513-4665)
Email address: lang_018@163.com

Supplementary Methods

Characterization and functional analysis of CF-EVs

The morphology and ultramicrostructure of CF-EVs are characterized by transmission electron microscope (TEM). The brief steps are as follows: add 10 μL EV suspension droplets to the Formvar carbon film copper net, leave them for 5 minutes, then dye with 2% phosphotungstic acid (pH 7.0) for 2 minutes, and then conduct TEM observation. Use the Zetasizer Nano ZS90 DLS system (Malvern Panalytical Company, UK) to determine the hydrodynamic diameter and Zeta potential of CF-EVs. The total protein concentration is quantitatively analyzed by the BCA protein test kit (Thermo Scientific Company of the United States).

Evaluation of free radical-scavenging activity

The antioxidant activity of CF-EVs was evaluated through a variety of free radical removal tests. ABTS+ free radical clearance activity is determined by monitoring the absorbance at 734 nm by a commercial kit; the hydroxyl free radical clearance activity is detected at 645 nm by the TMB method, while the DPPH free radical clearance activity is determined by measuring the absorbance change at 517 nm. The antioxidant ability is quantitatively analyzed according to the free radical removal efficiency. In addition, CIQTEK EPR200M spectrometer (CIQTEK Hefei, China) is used for electron paramagnetic resonance (ESR) spectral analysis to evaluate the removal effect of CF-EVs on ·OH and O2−. The EPR parameter settings are as follows: the central magnetic field is 3510 G, the scanning width is 500 G, the microwave power is 20 mW, and the modulation amplitude is 2 G.

Cellular uptake assay

CF-EVs are labeled with lipophilophic fluorescent dye Dil, and the free dye is removed by super-fast centrifugal purification. Incubate RAW 264.7 and Caco-2 cells with Dil-labeled CF-EVs at different times (1-24 hours), and then washed with PBS. Cell uptake efficiency is quantitatively determined according to the percentage of Dil-positive cells through flow cytometry. For fluorescence imaging, first fix the cell with 4% glutaraldehyde, then stain the cell skeleton with FITC-phosphatidylserine, stain the nucleus with DAPI, and finally observe under the Zeiss Axio Observer 5 fluorescence microscope.

Intracellular antioxidant assay

The intracellular antioxidant activity of CF-EVs was evaluated using the oxidative stress model induced by hydrogen peroxide. RAW 264.7 macrophages were pre-incubated at a concentration of 10 or 100 μg/mL for 12 hours, and then further stimulated with 400 μM H2O2 for 4 hours. DCFH-DA and DHE fluorescent probes were used to detect the accumulation of active oxygen (ROS) in cells and the generation of superoxide anions respectively. The change of fluorescence intensity is quantitatively analyzed by flow cytometry, and further visual observation is carried out by a confocal microscope.

Anti-inflammatory assay

Under the condition of inflammatory stimulation, the immunomodulatory effect of CF-EVs was studied. RAW 264.7 macrophages and Caco-2 cells were pretreated with different concentrations of CF-EVs (10 and 100 μg/mL) respectively and exposed to inflammatory stimulants. RAW 264.7 cells were activated with LPS (500 ng/mL) for 12 hours, while Caco-2 cells were first stimulated with TNF-α (30 ng/mL), and then induced oxidative stress with 600 μM H_2O_2 for 4 hours. After processing, the

total RNA was extracted, and the gene expression level associated with inflammation was determined by RT-qPCR analysis.

Quantitative real-time PCR

RT-qPCR technology was used to analyze the mRNA expression levels of pro-inflammatory cytokines (IL-6, IL-1β and TNF-α) in RAW264.7 cells, Caco-2 cells and colon tissues, as well as closely connected proteins (in Caco-2 cells and colon tissues ZO-1 and Occludin), and kidney injury-related markers in kidney tissue (KIM-1, NGAL, CCL-2 and ICAM-1). Total RNA is extracted through commercial kits and cDNA is generated by reverse transcription. RT-qPCR is tested using SYBR Green Master Mix in the LightCycler® 480 system produced by Roche in Switzerland. GAPDH is used as an internal parameter, and the relative gene expression is calculated by the $2^{-\Delta\Delta Ct}$ method. The sequence of the mimer is shown in Table S1.

Western blotting

The total protein is extracted using RIPA lysate containing protease inhibitors, and the protein concentration is determined by the BCA method. The same amount of protein is separated by 10% SDS-PAGE and transferred to the PVDF membrane. After blocking with 5% fat-free milk, the membrane is incubated overnight at 4°C, and specific antibodies to TLR4, NF-κB p65, phosphorylated p65 (p-p65), and β-actin are added. Then, incubate with HRP-labeled anti-incubation at room temperature for 1 hour. Use enhanced chemiluminescence (ECL) system to develop protein strips and quantify them by gel imaging analysis.

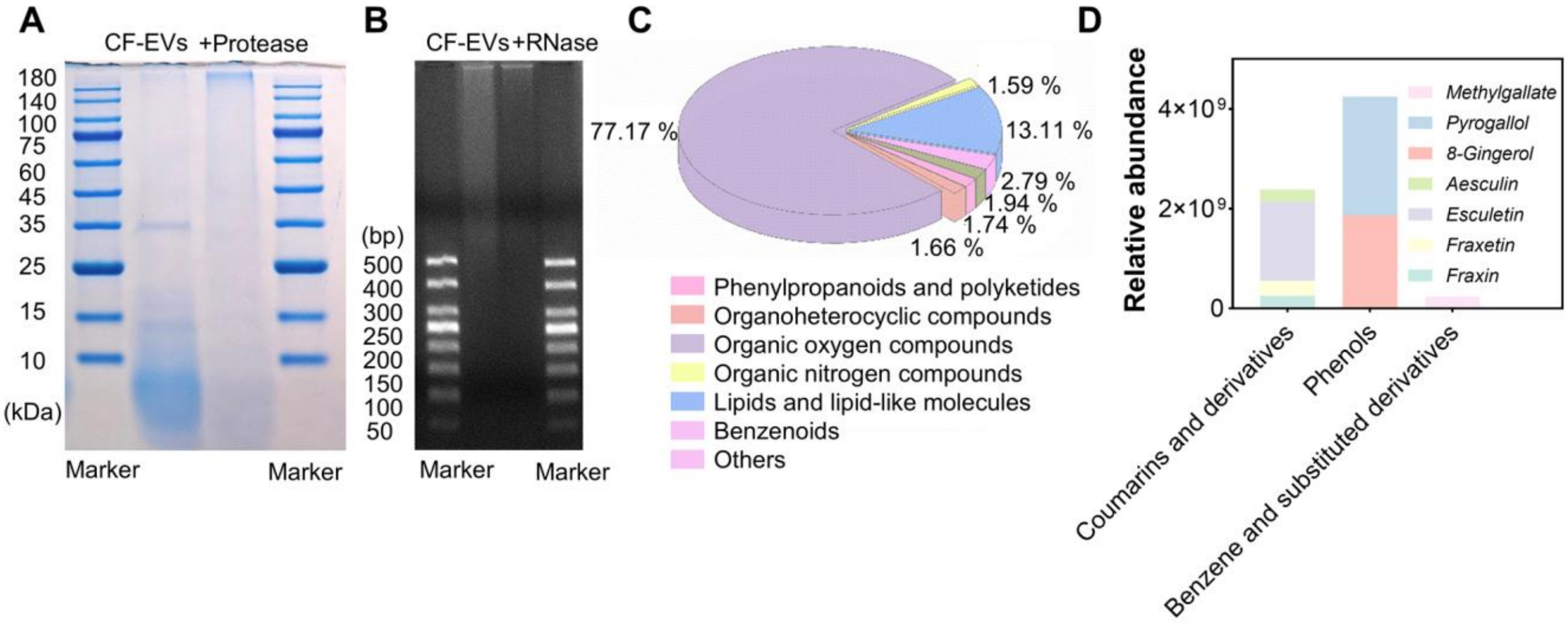

Figure S1. Characterization of molecular cargos and antioxidant-related metabolites in CF-EVs. (A) SDS-PAGE uses Coomassie blue staining to show the protein load carried by CF-EVs. (B) Agarose gel electrophoresis analysis shows that there is a nucleic acid load in CF-EVs. (C) Through non-targeted metabolomics analysis based on LC-MS/MS, the metabolic group distribution of compounds in CF-EVs was identified. (D) The relative abundance of representative bioactive plant chemicals detected in CF-EVs, including characteristic coumarin derivatives and phenolic compounds with antioxidant activity.

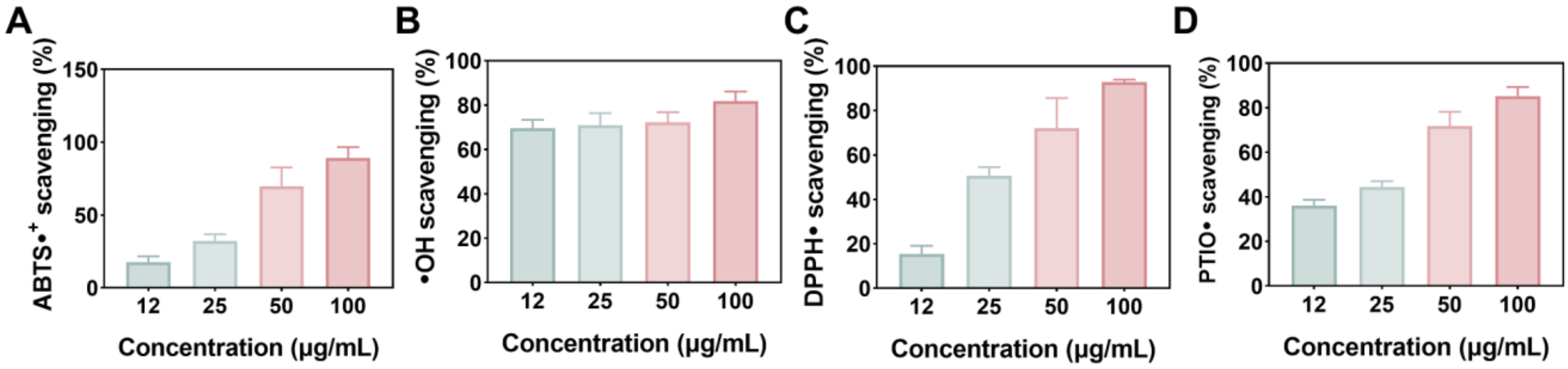

Figure S2. Antioxidant activity of CF-EVs@Inulin. (A–D) Evaluation of the antioxidant capacity of CF-EVs through multiple free radical-scavenging assays, including ABTS•+ (A), hydroxyl radical (•OH) (B), DPPH• (C), and PTIO radical (D) elimination assays. Data are expressed as mean ± SD (n = 3).

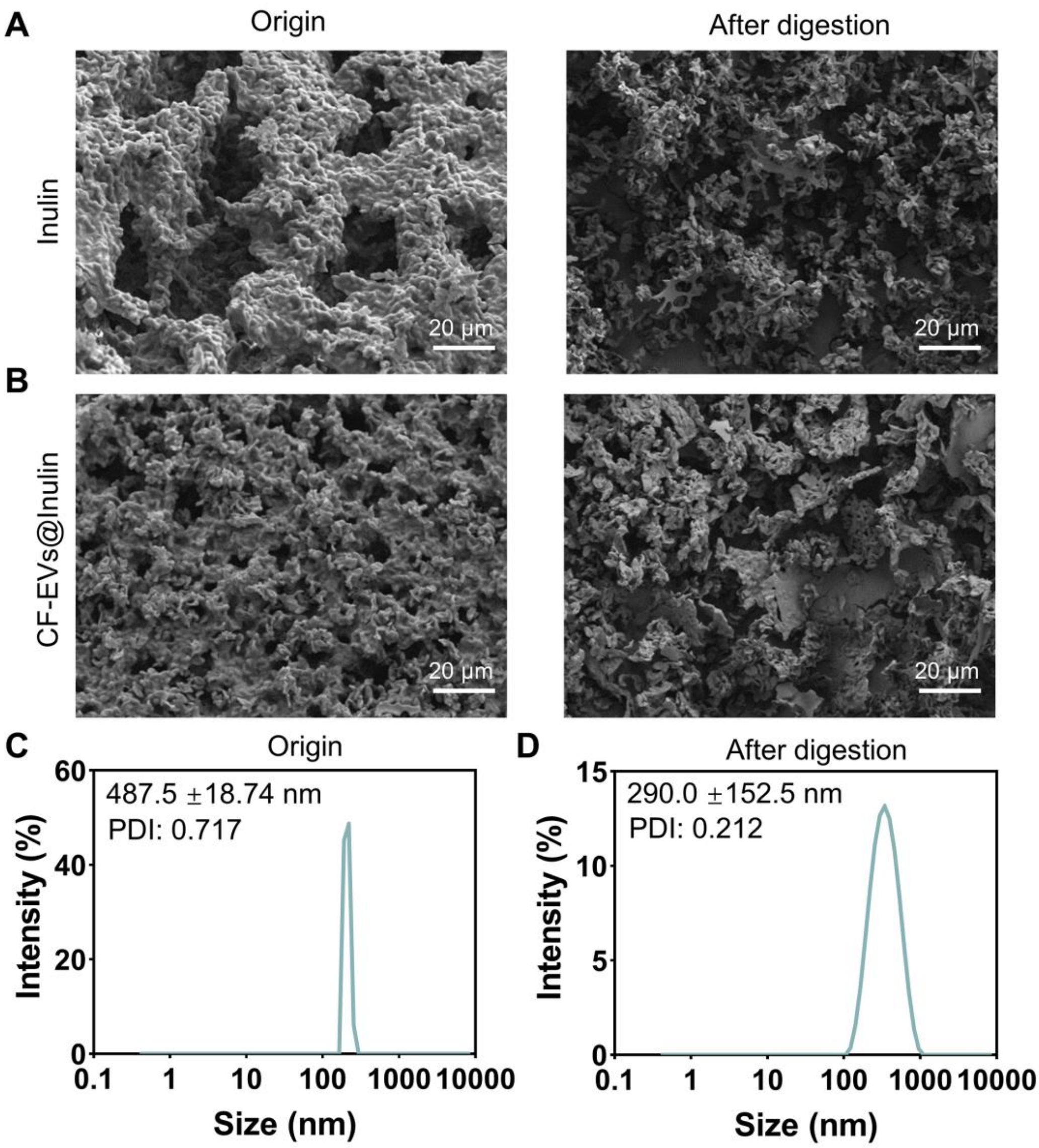

Figure S3. Characterization of CF-EVs@Inulin after simulated gastrointestinal digestion. (A) Morphological characterization of the inulin hydrogel following simulated gastrointestinal digestion by scanning electron microscopy (SEM). (B) SEM visualization of CF-EVs@Inulin after exposure to simulated gastrointestinal digestion conditions. (C–D) Hydrodynamic diameter distribution of CF-EVs released from the hydrogel system following simulated gastrointestinal digestion.

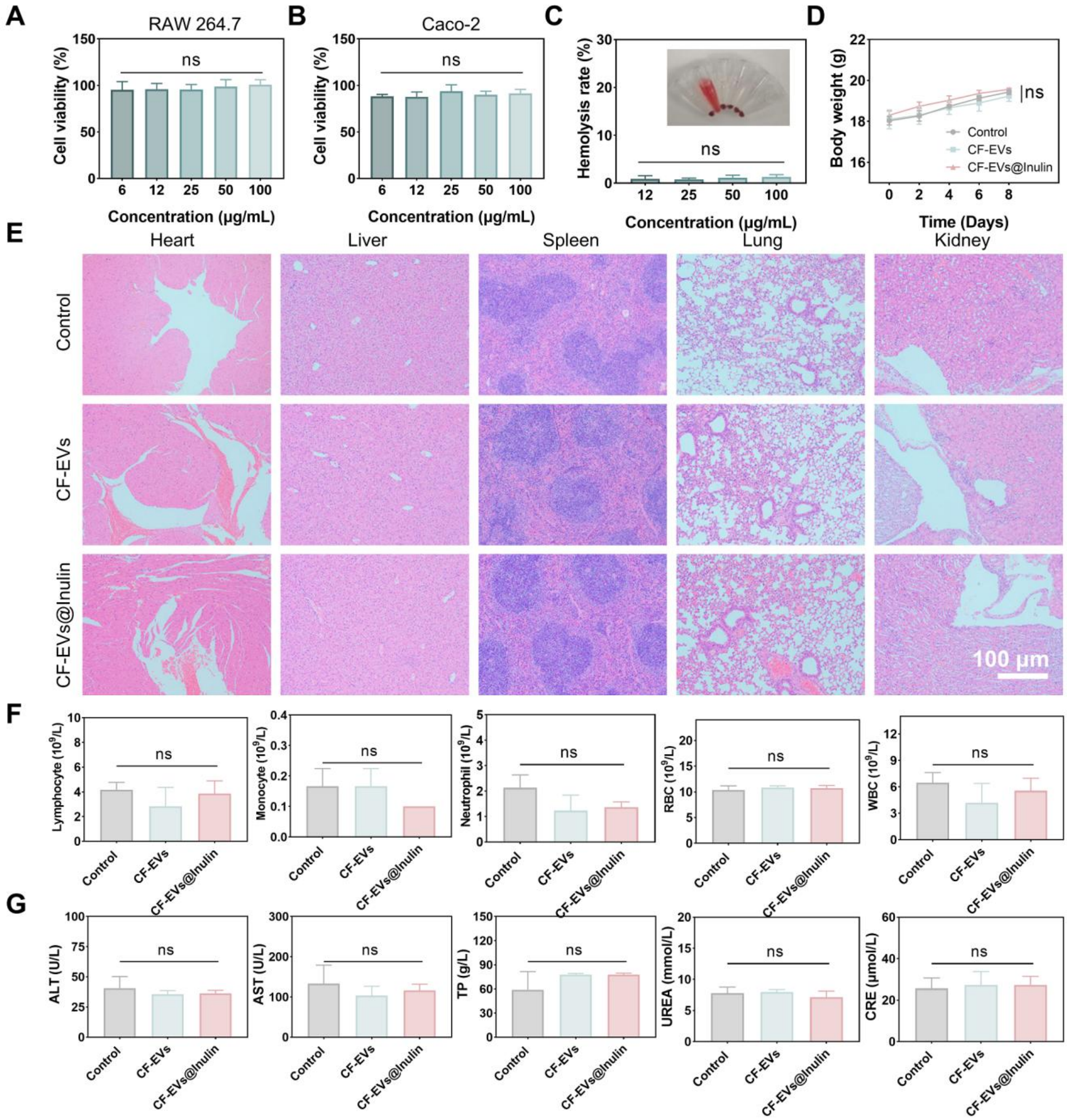

Figure S4. Biocompatibility and in vivo safety evaluation of CF-EVs and CF-EVs@Inulin. (A–B) Evaluation of RAW 264.7 and Caco-2 cell viability following treatment with different concentrations of CF-EVs using the CCK-8 assay. (C) Hemocompatibility assessment of CF-EVs and CF-EVs@Inulin based on hemolysis analysis. (D) Changes in body weight of mice administered CF-EVs or CF-EVs@Inulin by oral gavage for 7 consecutive days (n = 6). (E) Representative H&E-stained images of major organs, including the heart, liver, spleen, lung, and kidney. Scale bar: 100 μm. (F) Peripheral blood hematological parameters, including LYM%, Mono, NEU, RBC, and WBC levels (n = 3). (G) Serum biochemical indicators associated with hepatic and renal function, including ALT, AST, TP, UREA, and CRE (n = 3). Data are presented as mean ± SD. ns, not significant.

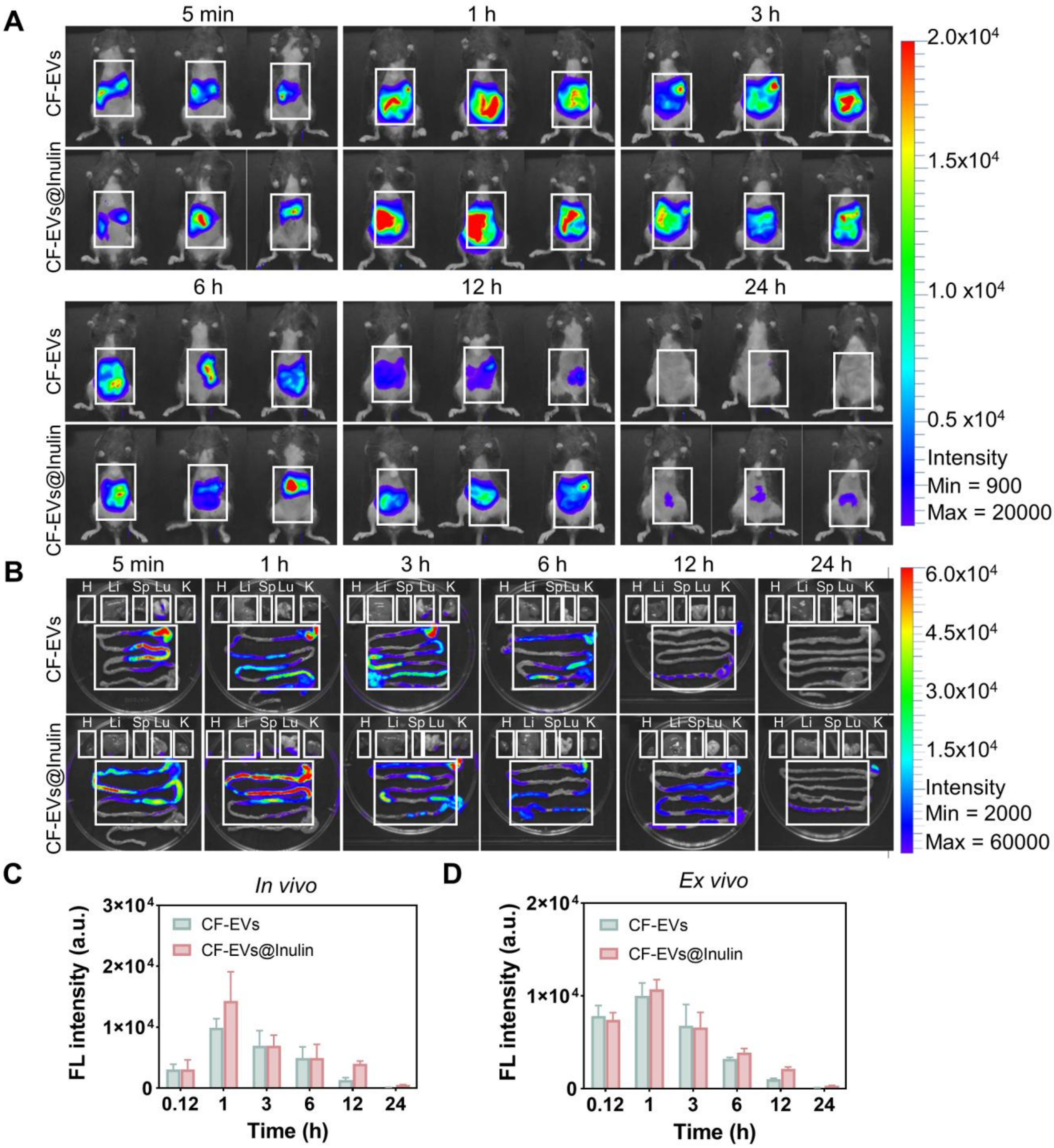

Figure S5. *In vivo* biodistribution of orally administered CF-EVs@Inulin. (A) In vivo fluorescence tracking of DiR-labeled CF-EVs and CF-EVs@Inulin following oral administration at different time points (5 min, 1 h, 6 h, 12 h, and 24 h). (B) Ex vivo fluorescence imaging of major organs collected at 24 h post-administration. (C) Time-dependent changes in whole-body fluorescence intensity quantified from IVIS imaging. (D) Quantification of fluorescence signals in isolated colon tissues, demonstrating enhanced colonic retention of CF-EVs following encapsulation within the inulin hydrogel. Data are expressed as mean ± SD, with representative images obtained from three mice per group.

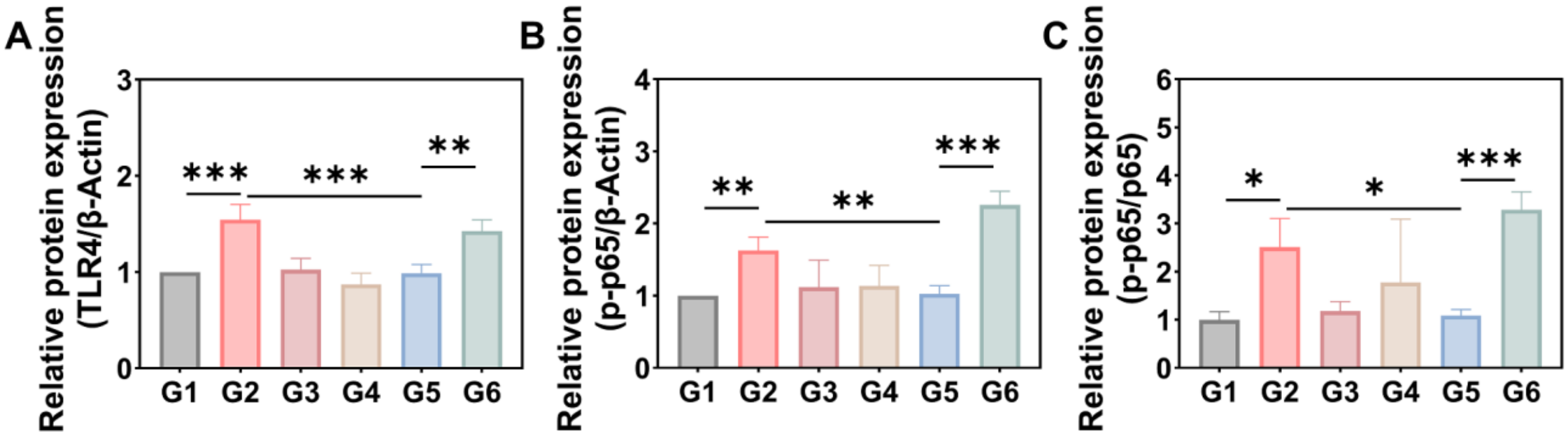

Figure S6. Quantitative analysis of Western blotting results is shown in Figure 10D. Relative expression levels of renal TLR4, p65, and phosphorylated p65 (p-p65) proteins were normalized against β-actin. (A) Densitometric analysis of TLR4 protein expression. (B) Quantitative evaluation of p-p65 protein abundance. (C) Analysis of the p-p65/p65 ratio as an indicator of NF-κB signaling activation. Data are presented as mean ± SD (n = 3). *$p < 0.05$, **$p < 0.01$, ***$p < 0.001$; ns, not significant.

Table S1. Primer sequences used for RT-qPCR.

Target gene	Forward primer (5'–3')	Reverse primer (5'–3')
IL-6 (Mouse)	ACAAGTCGGAGGCTTAATTACACAT	TTGCCATTGCACAACTCTTTTC
IL-1β (Mouse)	TCGCTCAGGGTCACAAGAAA	CATCAGAGGCAAGGAGGAAAAC
TNF-α (Mouse)	AGGCTGCCCCGACTACGT	GACTTTCTCCTGGTATGAGATAGCAAA
KIM-1 (Mouse)	CTGGAATGGCACTGTGACATCC	GCAGATGCCAACATAGAAGCCC
NGAL (Mouse)	ATGTCACCTCCATCCTGGTCAG	GCCACTTGCACATTGTAGCTCTG
CCL-2 (Mouse)	GCTACAAGAGGATCACCAGCAG	GTCTGGACCCATTCCTTCTTGG
ICAM-1 (Mouse)	AAACCAGACCCTGGAACTGCAC	GCCTGGCATTTCAGAGTCTGCT
ZO-1 (Mouse)	CCACCTCTGTCCAGCTCTTC	CACCGGAGTGATGGTTTTCT
Occludin (Mouse)	ATGTCCGGCCGATGCTCTC	TTTGGCTGCTCTTGGGTCTGTAT
GAPDH (Mouse)	TGTGTCCGTCGTGGATCTGA	TTGCTGTTGAAGTCGCAGGAG
IL-6 (Human)	ACTCACCTCTTCAGAACGAATTG	"CCATCTTTGGAAGGTTCAGGTTG"
IL-1β (Human)	ATGATGGCTTATTACAGTGGCAA	GTCGGAGATTCGTAGCTGGA
TNF-α (Human)	CAAAGTAGACCTGCCCAGAC	"GACCTCTCTCTAATCAGCCC"
Claudin-1(Human)	GTCTTTGACTCCTTGCTGAATCTG	CACCTCATCGTCTTCCAAGCAC
ZO-1 (Human)	GTCCAGAATCTCGGAAAAGTGCC	CTTTCAGCGCACCATACCAACC
Occludin (Human)	ATGGCAAAGTGAATGACAAGCGG	CTGTAACGAGGCTGCCTGAAGT
GAPDH (Human)	GTCTCCTCTGACTTCAACAGCG	ACCACCCTGTTGCTGTAGCCAA
